# Influenza virus segment 5 (+)RNA - secondary structure and new targets for antiviral strategies

**DOI:** 10.1038/s41598-017-15317-5

**Published:** 2017-11-08

**Authors:** Marta Soszynska-Jozwiak, Paula Michalak, Walter N. Moss, Ryszard Kierzek, Julita Kesy, Elzbieta Kierzek

**Affiliations:** 1 Institute of Bioorganic Chemistry Polish Academy of Sciences, 61-704 Poznan, Noskowskiego 12/14 Poland; 2 Roy J. Carver Department of Biophysics, Biochemistry and Molecular Biology, Iowa State University, Ames, IA 50011 United States of America

## Abstract

Influenza A virus is a threat for humans due to seasonal epidemics and occasional pandemics. This virus can generate new strains that are dangerous through nucleotide/amino acid changes or through segmental recombination of the viral RNA genome. It is important to gain wider knowledge about influenza virus RNA to create new strategies for drugs that will inhibit its spread. Here, we present the experimentally determined secondary structure of the influenza segment 5 (+)RNA. Two RNAs were studied: the full-length segment 5 (+)RNA and a shorter construct containing only the coding region. Chemical mapping data combined with thermodynamic energy minimization were used in secondary structure prediction. Sequence/structure analysis showed that the determined secondary structure of segment 5 (+)RNA is mostly conserved between influenza virus type A strains. Microarray mapping and RNase H cleavage identified accessible sites for oligonucleotides in the revealed secondary structure of segment 5 (+)RNA. Antisense oligonucleotides were designed based on the secondary structure model and tested against influenza virus in cell culture. Inhibition of influenza virus proliferation was noticed, identifying good targets for antisense strategies. Effective target sites fall within two domains, which are conserved in sequence/structure indicating their importance to the virus.

## Introduction

Influenza is a common disease that spans the globe and is dangerous for human health and life. Even seasonal flu is life threatening for young infants, the elderly, and immunocompromised patients. The most dangerous outbreaks are global pandemics that appear sporadically when immunologically novel influenza strains (e.g. from animal populations) gain the ability to infect humans, causing severe illness and complications in healthy populations.

Influenza is a single-stranded negative sense RNA virus with segmented genome (viral RNA - vRNA). The vRNA genome of influenza is divided into eight segments, which are independent transcription-replication units. Each step in the influenza life cycle is dependent on RNA. vRNA is used to generate two types of (+)RNA: cRNA (complementary RNA), and mRNAs (messenger RNA). cRNA is used as a template for replicating vRNAs while mRNA encodes viral proteins. More than 8 viral proteins are encoded through alternative splicing and translation initiation. Recent studies are revealing the roles of vRNA and mRNA secondary structure during different steps in the viral life cycle^1,2^. Knowledge about secondary structure provides insights to better understand virus biology and could be used for designing new therapeutics; however, additional work is needed to experimentally model influenza structure.

Antisense oligonucleotide (ASO) drugs are designed to alter RNA expression and function, thus allowing one to influence pathological processes beyond protein or receptor involvement^3^. Oligonucleotides are a major focus of drug development; numerous clinical trials have been performed and many oligonucleotide therapeutics are in phase III development. Several oligonucleotide therapeutics are FDA approved^4,5^. Oligonucleotide strategies offer advantages in comparison to other existing approaches against influenza. In fast evolving viruses, such as influenza, targeting surface proteins is challenging, as each season novel proteins appear in circulating strains. In contrast, the RNA of influenza, due to combination of evolutionary constrains, possess features that remain constant between strains. One such feature is the need of influenza to conserve RNA secondary structure. Therefore, viral RNA secondary structure determination is a key component in the design of effective inhibitory oligonucleotides.

Bioinformatics studies showed the possibility of conserved structures both in (+) and in (–) RNA strands^6,7^. Only several of these motifs have been examined experimentally, and very few have been studied *in vivo* ^2,8–10^. Only for two entire viral genomic segments, 8 and 7, has secondary structure been determined^11,12^. The structural information derived for segment 8 was used for designing effecting antisense oligonucleotides (ASOs) that inhibited virus proliferation^13^. Influenza RNA possesses complex and partially dynamic secondary structure, and the *in vitro* studies determining the conserved structure of vRNA elements bring us closer to interpreting their functions. In the case of mRNA, conserved structural motifs were discovered using sequence/structure comparisons, an analysis of the constraints of RNA folding on codon evolution, and were confirmed by the study of RNA models *in vitro* in segment 8, 7 and 5^8,9,14,15^ and experiments *in vivo* ^2^.

In this current study, we focus on the (+)RNA of segment 5. The segment 5 mRNA differs from cRNA by being capped and polyadenylated. Segment 5 mRNA encodes nucleoprotein (NP), which is a structural protein that also takes part in the regulation of transcription and replication^16^. NP, along with the viral polymerase and vRNAs, create eight viral ribonucleoprotein complexes (vRNPs), which are organized into an active virus particle. The crucial role of NP in virus assembly and the viral replication cycle makes segment 5 an attractive target for screening effective inhibitors. Three phosphorothioate modified antisense oligonucleotides targeting NP gene significantly inhibited H5N1 type virus replication; one oligonucleotide could protect the mice from a lethal dose of influenza virus^17^. Recently, a 121 nt conserved structural motif from the segment 5 (+)RNA was used to design effective antisense oligonucleotides against a modified, single-cycle infectious influenza A virus strain. This highlights the importance of this motif in virus proliferation and also highlights a new method for the design of ASOs based on target RNA secondary structure^8^. Segment 5 (+)RNA was also the focus of other studies, where additional predicted motifs in other regions (nts 16–39, 89–105, 577–593, 922–938, 1476–1530) were suggested to be important^18^. Therefore, segment 5 (+)RNA, which is rich in predicted structural motifs, was selected for the full-length analyses of (+)RNA structure: to validate/model these motifs in the context of the full-length sequence.

Herein, two RNA constructs were studied: the full-length segment 5 (+)RNA5 and a shorter construct, (+)RNA5-ORF, containing only the open reading frame (ORF) for NP. Here, (+)RNA5 corresponds to the cRNA generated from vRNA as well as the mRNA (minus the cap and poly(A) tail). Influenza generates mRNA through a “cap snatching” mechanism, where fragments of host mRNA 5′ untranslated regions (UTRs, including caps) are stochastically cleaved and extended to contain the viral (+)RNA sequence; the poly-A tail is added during mRNA maturation. (+)RNA5-ORF is the (+)RNA minus flanking viral UTR sequences and was included to assess the impact of removing the partially complementary UTRs, which in the vRNA are used to circularize the molecule (via a vRNA “panhandle” structure^19,20^) and in the (+)RNA can form a cRNA panhandle formed by the vRNA reverse complement sequence. Due, however, to vRNA GU pairs being converted to CA mismatches in the cRNA, this cRNA panhandle is structurally distinct. This structural distinction between the vRNA and cRNA panhandle structures is proposed to play a key role in regulating the promoter site used for cRNA, vRNA and mRNA replication^21^. Based on experimental data and bioinformatics analysis, the secondary structure for entire (+)RNA5 is proposed here. This is the first experimentally informed secondary structure model of (+)RNA5, which presents features likely to be important in segment 5 mRNA or/and cRNA function. This structure model was used to design antiviral oligonucleotides, which were shown to have activity in inhibiting the native influenza virus replication in an MDCK cell line.

## Results

### Chemical mapping of segment 5 (+)RNA

**Figure 1:**
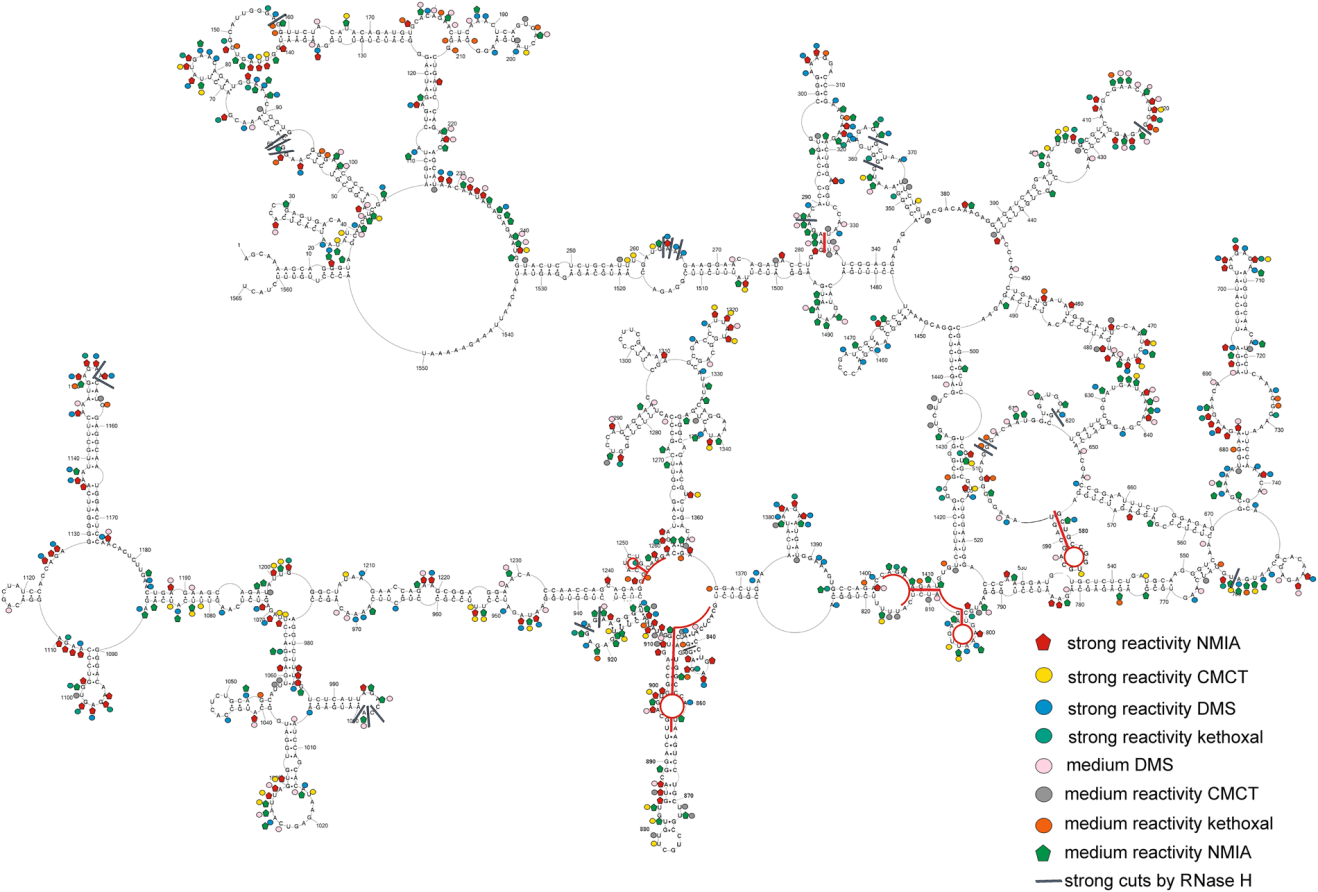
Self-folding of (+)RNA5 predicted by RNAstructure 5.5 using experimental data obtained in 23 °C as constraints. Strong DMS, CMCT and kethoxal modifications, as well as SHAPE reactivities converted to pseudo-free energies were used. Additionally medium reactivity DMS, CMCT, kethoxal and results from RNase H cleavage in presence of DNA oligonucleotides (neither used in modeling) are also annotated. Red lines indicate regions of structure that differ at higher temperature (37 °C). The numbering of (+)RNA5 is from its 5′ end. The AUG start codon spans nucleotides 46–48.

**Figure 2:**
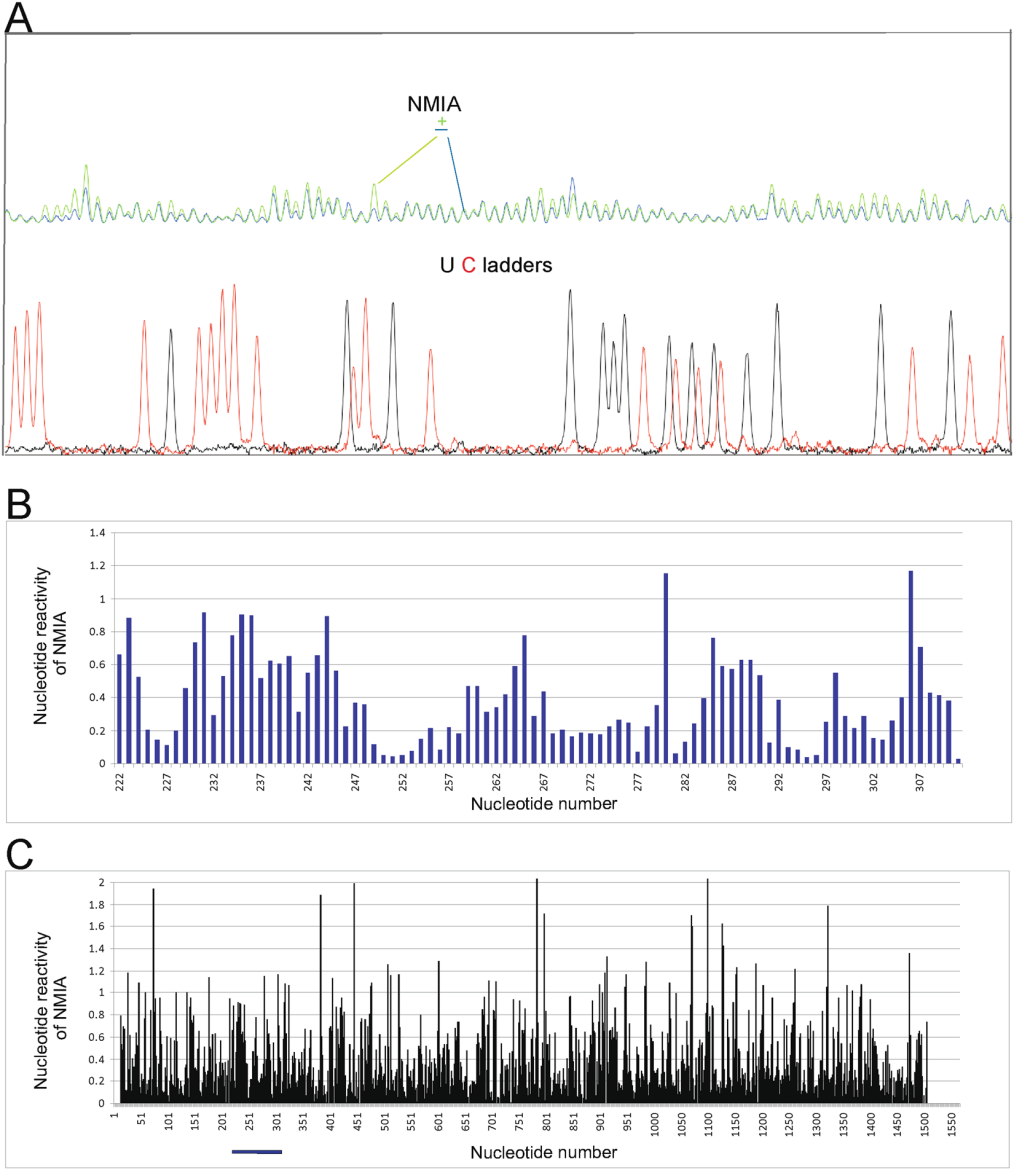
Chemical modifications data at 23 °C with NMIA for (+)RNA5 detected by reverse transcription with labeled primers followed by capillary electrophoresis. (**A**) Example of capillary electrophoresis raw data for nt 311–222 showing modified RNA (dark green line), unmodified control (light green line) and dideoxy ladders (C – red line and U – black line). (**B**) (+)RNA5 nt reactivities from SHAPE for the same region showed in panel A, in reverse order (222–311 nt) on the graph. (**C**) (+)RNA5 nt reactivities for SHAPE across the entire (+)RNA5. The nt region (222–311) showed on panel B is marked by line.

CMCT (modifies N3 of U and N1 of G when unpaired), DMS (methylates N1 of A and N3 of C when unpaired), and kethoxal (modifies G when unpaired)^22,23^ and SHAPE mapping (modifies all flexible, e.g. single stranded, bases)^24^ were applied to probe the folding in (+)RNA5 and (+)RNA5-ORF. Before mapping, appropriate folding conditions were found to yield a single RNA conformation, as assessed by non-denaturing agarose gel. Both (+)RNA5 and (+)RNA5-ORF adopt a single conformation in buffer containing 100 mM KCl (or 300 mM) and 5 mM MgCl_2_. Mapping was carried out at 23 °C and 37 °C to assess the impact of temperature on folding. This range of temperatures was used because there are influenza strains that can effectively replicate in low temperature (e.g. cold adapted viruses) and others in high temperature (e.g. avian strains)^25,26^; it is thus valuable to assess how RNA folding may be affected by different temperatures. To read out mapping results, reverse transcription was used with fluorescent labeled primers (Supplementary Table S2) followed by capillary electrophoresis. The analyzed RNAs undergo selective modifications, showing evidence of these being structured RNA molecules. There are relatively short regions that show reactivity at 23 °C (Figs 1, 2, Figures S2–S4, Supplementary Data 1). There are some small differences in reactivity at 37 °C, but they do not significantly alter the general reactivity pattern (Supplementary Data 1).

### Circular folding of segment 5 (+)RNA

To predict the secondary structure of segment 5 (+)RNA based on the experimental probing data, the results of chemical mapping were used to constrain predictions in the RNAstructure 5.5 program. SHAPE data were loaded as pseudoenergy constraints (the energy contribution of SHAPE reactive nt were penalized) and DMS, CMCT, and kethoxal modifications were included as chemical mapping constraints (reactive nt are forbidden to be in Watson-Crick base pairs flanked by Watson-Crick base pairs). Additional constraints were added to constrain pairing in previously-modeled conserved structural motifs in regions: 1051–1171 nt (1077–1197 (+)RNA5-ORF) pairs 1081/1191, 1083/1190 and 1085/1189 ((+)RNA5 numbering) were added as base pairing constraints^8^.

The secondary structure of (+)RNA5 appears circular in folding (Fig. 1). Several long distance interactions exist in (+)RNA5: helixes 6-14/1560-1551, 244-281/1533-1497, 497-523/1446-1414. The long-range helix (6-14/1560-1551) recapitulates the cRNA panhandle structure previously determined by NMR^27^. There are distinct structural motifs that are well defined by chemical mapping, such as the hairpins in regions: 45–105, 283–334, 388–444, 451–492, 671–743, 1129–1173. SHAPE and chemical mapping show that there are fourteen relatively long flexible regions (each containing at least 7 flexible nucleotides in sequence: 73–79, 141–148, 231–239, 261–265, 283–289, 313–319, 414–426, 470–477, 633–639, 683–687, 756–762, 907–913, 1024–1031, 1096–1102 nt).

Possible changes of secondary structure at higher temperature (37 °C) were assessed due to fact that influenza can propagate in different temperatures in various species. The model secondary structures of (+)RNA5 probed at 23 °C and 37 °C are almost identical and only minor changes were noticed (Supplementary Figure S5, Supplementary Data 1). Folding of only four regions (283–290/328–334, 577–593, 797–815/1403–1410, 833–907/1245–1256) was different (Supplementary Figure S5, Fig. 1). These data showed that (+)RNA5 is globally stable at elevated temperatures and only four regions appear to be labile.

### Local structural motifs in (+)RNA5 are mostly independent of long range interactions

To test if UTR interactions can influence local folding within the coding region, the structure of a coding-region only construct, (+)RNA5-ORF, was determined. The modeled structure was almost identical to that of corresponding coding region of (+)RNA5, indicating that local motifs within the ORF are stable independently of the UTR fold. Only the structures of two regions (577–593, 833–931) were different (Figure S6 and Fig. 1). Thermodynamically stable local motifs appear in the (+)RNA5-ORF secondary structure. For example, in the (+)RNA5-ORF model, hairpin 532–545 ((+)RNA5-ORF numbering, Supplementary Figure S6) has predicted ΔG°_37_ = −6.2 kcal/mol, whereas in entire (+)RNA5 hairpin 577–593 (Fig. 1) is predicted to be less stable (ΔG°_37_ = −4.6 kcal/mol). Similarly, domain 832–931 of (+)RNA5-ORF (Supplementary Figure S6, entire (+)RNA5 numbering) is folded into a more thermodynamically stable structure with ΔG°_37_ = −30.3 kcal/mol comparing to (+)RNA5 structure with ΔG°_37_ = −27.0 kcal/mol (Fig. 1). It is possible that the existence and appropriate folding of 5′ and 3′ ends has some, but relatively small, influence on global folding of (+)RNA5.

The possibility of preferential local folding in segment 5 (+)RNA was also considered for the entire (+)RNA5. For that purpose, beside experimental constraints, a maximum paring distance of 600 nt was set in RNAstructure 5.5 during the prediction of secondary structure: here, the greatest allowable distance between base pairs is 600 nt. All other experimental constraints were also incorporated in this calculation. The consequences of this additional constraint are the disappearance of helix 244–281/1478–1533 and the appearance of several new motifs on the 5′ and 3′ ends (Supplementary Figure S7). Besides these few novel folds, the remaining motifs are identical as the calculation without the 600 nt distance constraint.

### Base pair probabilities

**Figure 3:**
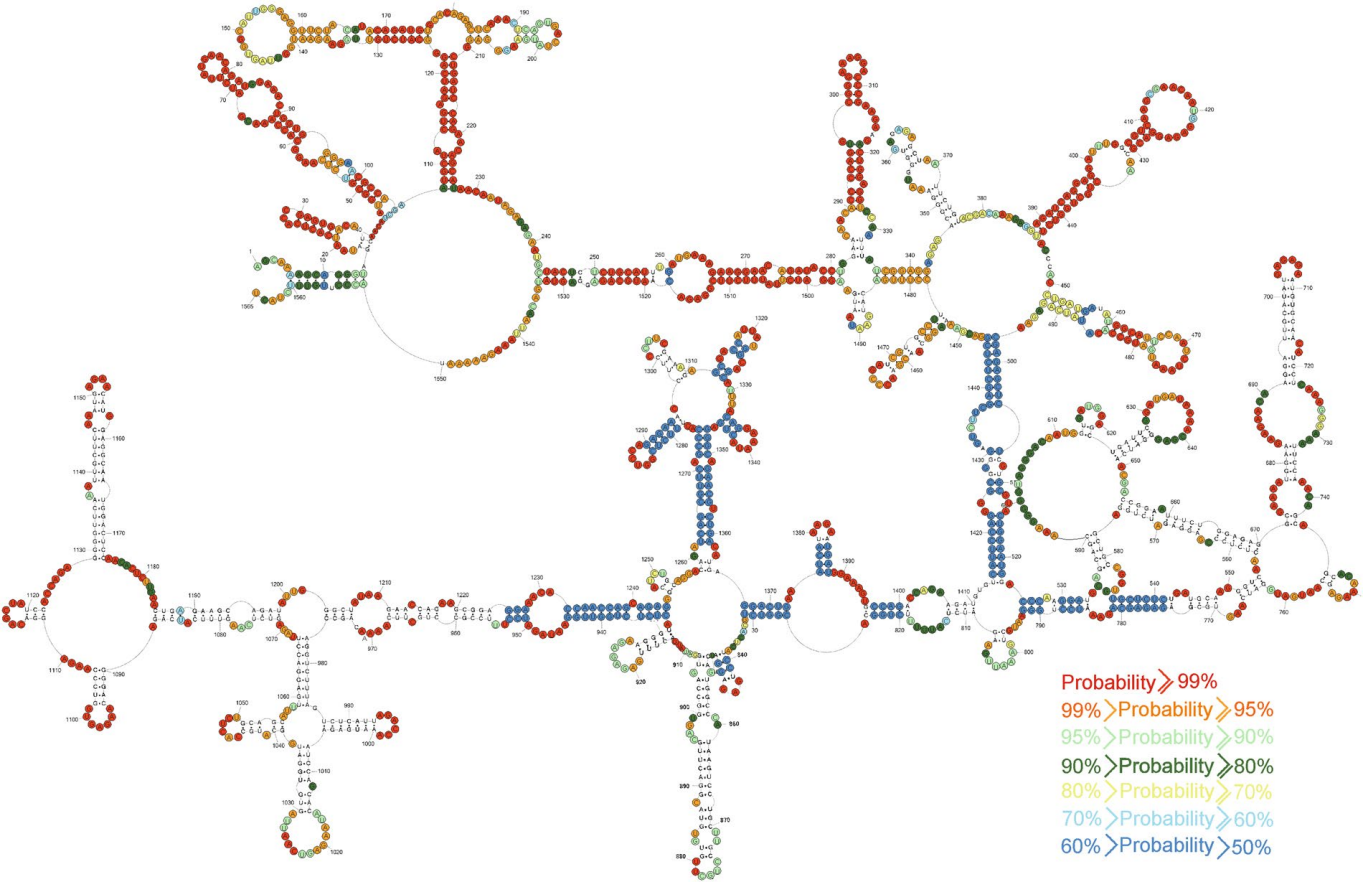
Predicted probability of nt being paired or single stranded in (+)RNA5 using RNAstructure program. Probability lower than 50% is not colored. The partition function calculation incorporated restraints from strong reactivity of DMS, CMCT, kethoxal and SHAPE (converted to pseudo-energy).

To assess prediction quality and identify well-defined structural regions we calculated the secondary structure partition function using RNAstructure 5.5 and, from this, determined the base pair probabilities for model pairs^28^. For the partition function calculations, experimental data were included. Results indicate that there are several regions with paired and unpaired nucleotides of more than 80% probability: 1–342, 388–492, 1480–1565 (Fig. 3). Additionally, all single stranded regions are well defined by having low probability of pairing.

### Conservation of (+)RNA5 model structure throughout influenza A strains

**Figure 4:**
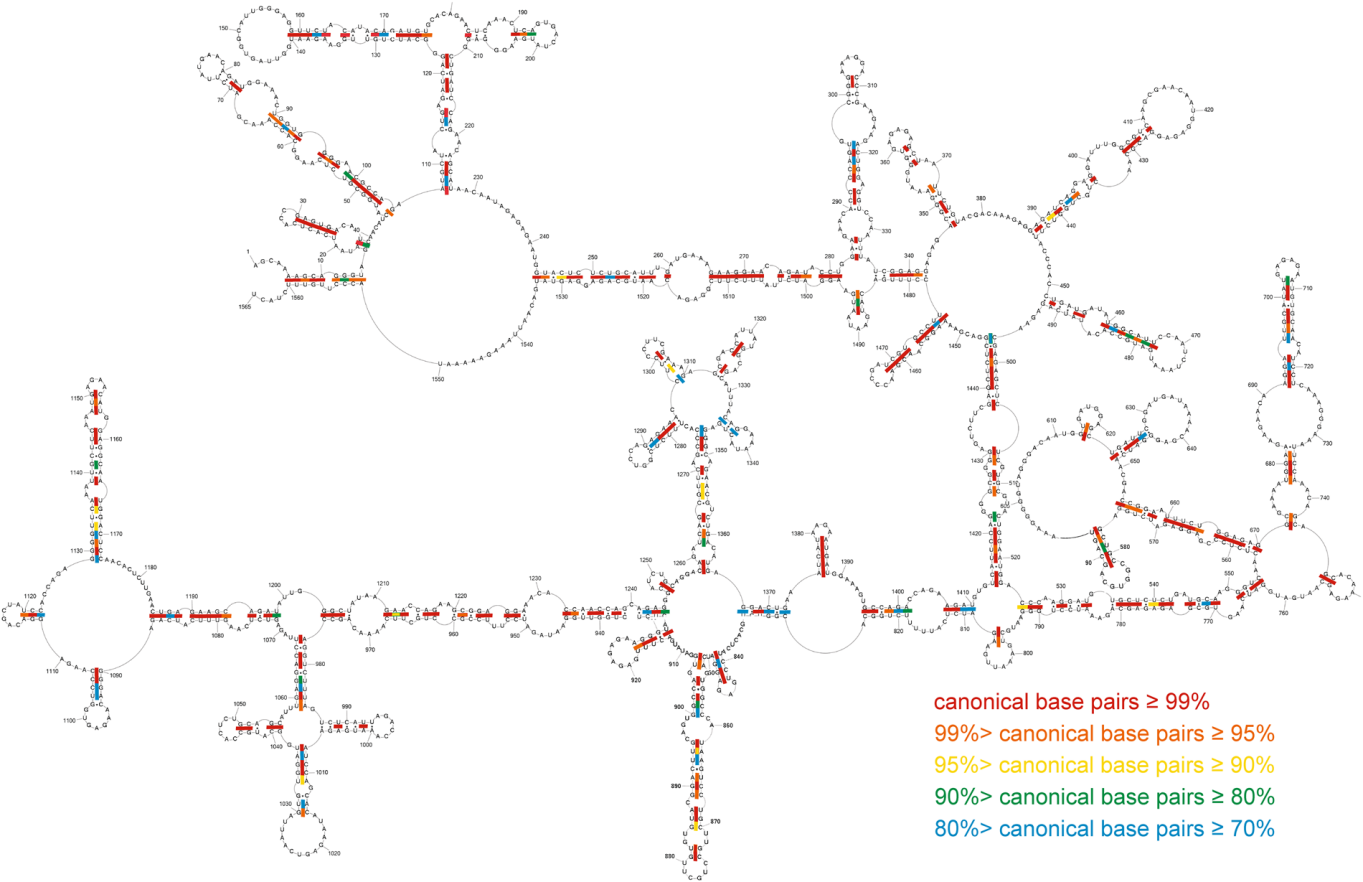
Conservation of (+)RNA5 secondary structure in type A of influenza viruses. Colors indicate percentage of canonical base pairing preserved across influenza A sequences for (+)RNA segment 5.

15533 unique sequences of (+)RNA5 were acquired from the NCBI Influenza Virus Resource^29^. Aligned sequences were analyzed with respect to the experimentally constrained (+)RNA5 structure model to identify the level of base pair conservation and identify structurally relevant mutations. Overall, model base pairs are 85% conserved across all analyzed sequences (measured as the percentage of canonical pairs [GC, AU and GU] observed in the alignment) (Fig. 4). The most common pairs are GC pairs, followed by AU pairs in roughly similar amounts (37.7% and 34.6%, respectively; Supplementary Data 2). After Watson-Crick pairs, GU pairs are the most commonly observed (13.3%) followed by CA pairs (6.9%) and others in much lower abundance.

The constraint of codon evolution in much of this sequence makes the identification of statistically-significant compensatory mutations difficult; however, we can assess the mutual information (MI), to suggest co-evolution of paired nt in the alignment^30^. The only extended regions with higher-than-average MI (Supplementary Data 2) were the noncoding nt that form the cRNA panhandle (nt 6–14/1560–1551) and the short hairpin between the panhandle (17–26/38–30) and NP start codon (Fig. 1), which are free of the constraint of maintaining codons.

### Hybridization to Isoenergetic Microarrays – oligonucleotide accessible regions

(+)RNA5 accessibility to oligonucleotides was studied using isoenergetic microarrays and RNase H cleavage induced by DNA oligonucleotides^31–33^. Short penta- and hexanucleotide probes that are 2′-O-methyl-RNA with LNA and 2,6-diaminopurine modifications at selected positions were used to build isoenergetic microarrays. Hybridization of duplex probe/RNA are engineered to have near equal free energies^34–36^; thus, only the secondary structure of the RNA should influence binding of these isoenergetic probes, not the sequence-specific stability of duplex formation^31–33^. Hybridization of (+)RNA5 to the isoenergetic microarray was conducted at 23 °C and 37 °C in buffer containing 300 mM KCl, 5 mM MgCl_2_ and 50 mM HEPES pH 7.0. Only strong and medium binding probes were considered in the analysis of structure (Tables 1 and S4, Figure S9).

**Table 1:** Deduced strong and medium binding sites in (+)RNA5 for microarray probes.

Binding site^a,b^	Confirmed binding sites^b,c^	Strength of probe binding^d^	Probe sequence^e^	Predicted ΔG°_37_ of probe/(+)RNA5 duplex (kcal/mol) for confirmed binding sites^f^	RNase H cleavage sites
154/1202	154	M	CcCdA	−8.23 (154)	157
155/791	155	S	uCcCa	−8.86 (155)	157
156/207/1430	156	S	CUcCc	−9.16 (156)	157
264	264	M	UcUuUcA	−9.12 (264)	262–264
13/601/930	601	M	UdCcCg	−9.85 (601)	604–605
365/501/753/778/1311	365	M	GcUcUg	−10.26 (365)	365
237/344/364/366/424/500/668/705/775/777/845/921/923	424/845/921/923	S	CUcUcg	−9.08 (424/845/921/923)	422/847/924–926
343/389/423/568/667/1515	423	S	UcUcCg	−9.81 (423)	422/847/924–926
386/425/669/846/1089/1110/1128/1243/1526	425 846	M	CcUcUg	−10.09 (425, 846)	422/847/924–926
341/387/398/426/, 566/847/1062/, 1111/1225/1367/1527	426 847	M	uCcUcg	(−9.99) 426 (−9.99) 847	422/847/924–926
206/605/644/728/1091	644	S	UcCcUg	−10.47 (644)	642
645	645	S	AuCcCg	−9.11 (645)	642
880/1556	880	S	DcDdGg	−7.73 (880)	880

^a^possible complementary binding sites of probes, ^b^sites are denoted by the middle nucleotide of the complementary RNA region (or two nucleotides for probes with an even number of nucleotides); ^c^deduced binding site for probe by comparison with DNA induced RNase H cleavage results; ^d^binding was considered strong (S), medium (M) and weak (W), when the integrated intensities were ≥1/3, ≥1/9 and ≥1/27 of the strongest intensity. Hybridization condition: buffer 300 mM KCl, 5 mM MgCl_2_, 50 mM HEPES, pH 7.0, 37 °C; ^e^nucleotides in capital letter (A, C, G, U, D) are 2′-O-methyl-RNA nucleotides, lower case letters (a, c, g, u, d) are LNA nucleotides, D and d are 2,6-diaminopurine (2′-O-methyl type or LNA, respectively); ^f^ΔG°_37_ calculated as modified probe/RNA duplex^35,36^, in parenthesis, the site of binding for which calculation was done.

**Figure 5:**
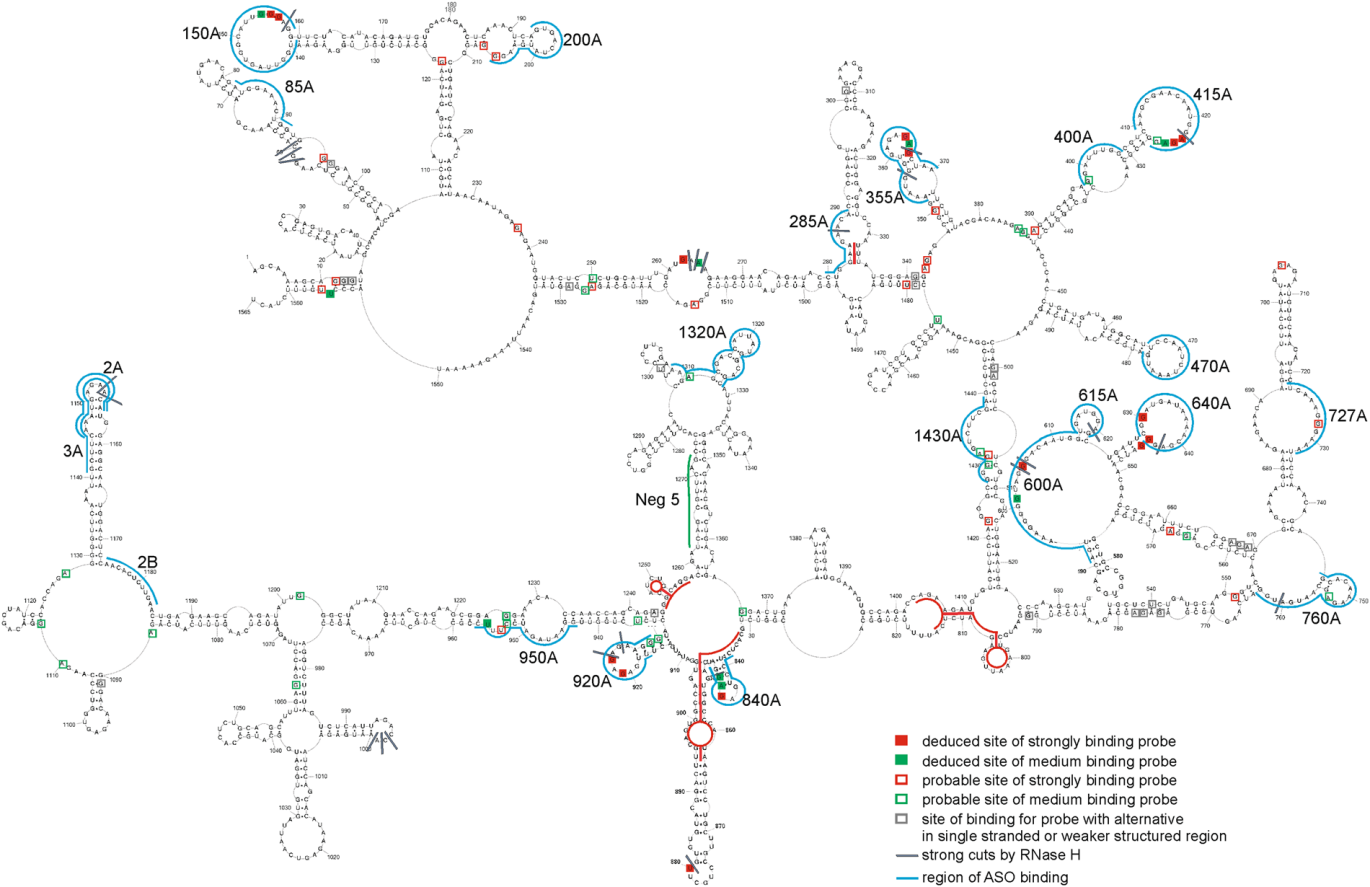
(+)RNA5 accessibility to oligonucleotides. Results from microarray mapping and RNase H cleavage in presence of DNA oligonucleotides at 37 °C are annotated. Additionally, target regions for designed ASOs are showed in the context of (+)RNA5 structure. Binding sites of pentamer probes are denoted by the middle nucleotide of the five nucleotides complementary in the RNA. In case of hexamer probes, binding sites are denoted by the fourth complementary nt in the RNA. Site 264 binds a heptamer and the middle nt of the seven nt complementary in the RNA is marked. The red line indicates regions of structure difference upon higher temperature (37 °C).

RNase H cleavage was used to validate several oligonucleotide probe binding sites (Figs 1 and 3, Supplementary Figure S8). 19 DNA oligonucleotides were used to test accessibility of the (+)RNA5 regions identified by microarray mapping. Generally, RNase H cleavages were observed at the expected single stranded regions: after nucleotides 157, 287, 356, 365, 422, 604–605, 620, 642, 760, 1153, and 1154 (Fig. 5 and Supplementary Table S5). Additional sites in single stranded regions: 58–60, 923, 925, 997–999 were cleaved by RNase H, showing that these DNA oligonucleotides also bind partially in these regions. Cleavages were also observed after 262–264 (internal loop) and 847 nt. Cleavage after 847 was within a helix, but DNA oligomer that induced that cleavage, CTCTCCATT, is complementary to a helix/loop region. Weak cleavages were observed in single stranded regions after 473, 705, 729, 880 and 1181. Nucleotides 1060-1068 are sequestered in a helix interaction, and were not accessible to complementary oligomer AAGGTCCTC. Oligonucleotides CAAATCCTC and GGATCTATT did not cause cleavage (complementary to regions: 396–404, 943–953, respectively), even though (in proposed secondary structure) complementary regions are single stranded; this suggests the possibility of tertiary, long distance interactions, occurring in these probe-inaccessible regions.

The probe binding sites: deduced (confirmed by RNase H cleavage) and probable (assigned after analysis of the most possible alternative binding sites in terms of hybridization duplex free energy and target structure) were marked on the (+)RNA5 secondary structure (Fig. 5 and Table 1, Supplementary Table S5). The binding of modified probes are almost identical in 23 °C and 37 °C. Only one probe, AcCcUg (nt in capital letters are 2′-O-methyl-RNA nucleotides, while lower case letters indicate LNA nucleotides) differs in binding intensity to (+)RNA5: it binds strongly in 23 °C and with medium intensity at 37 °C. Accessible sites: 154, 155, 156, 264, 365, 601, 605, 630, 845, 880, 921, 923 are in single stranded regions (Fig. 5). Binding to the microarray was also observed at regions closing a hairpin loop or internal loop: sites 644, 645, 846, 956. Generally, helical regions of (+)RNA5 were not accessible for probes. Additionally, several predicted hairpin loops, for example 467–477 and 1016–1029, are not accessible to probes. Tertiary structure and local non-canonical interactions could be affecting binding in these hairpins.

### Inhibitory effect of oligonucleotides targeting segment 5 (+)RNA in cell culture

The highly conserved (across type A influenza strains) secondary structure of segment 5 (+)RNA presented in this work allow to designate new targets for oligonucleotides. The new target regions, could be alternative to those previously found for the NP (+)RNA^8,17^ and confirmation of determined secondary structure elements. Antisense oligonucleotides (ASOs) were designed to be fully complementary to segment 5 (+)RNA of strain A/California/04/2009 (H1N1) (Table 2). All synthesized and tested ASOs were 2′-O-methyl RNA with LNA nucleotides to stabilize binding to target regions. ASOs were designed to target single stranded regions that we found to strongly bind microarray probes and/or are well-defined as single-stranded (based on the chemical mapping of segment (+)RNA5) (Fig. 5). Two control ASOs were also used: oligonucleotide N^17^ (sequence nonspecific to influenza virus RNA) and Neg 5 (complementary to a region of (+)RNA5 that forms a helix: 1262-1272) (Table 2, Fig. 5). The nucleotide sequence conservation of each site was calculated (Table 2). The average nucleotide sequence conservation for the entire segment 5 (+)RNA is 87.6% and 89.7% for ORF. Average nucleotide sequence conservation for ASO binding sites is slightly higher than the overall conservation: 90.1% (ranging between 85.1% and 95.2%).

**Table 2:** ASOs targeting (+)RNA5.

Name	Length (nt)	Sequence 5′ → 3′^a^	Complementary (+)RNA5 region^b^	Conservation in target region nucleotide sequence^c^ (%)
1430A	17	CGaAGAcUCCcCGcCCC	1427–1439	97.9
85A	11	CCaGUcUCcAU	82–92	94.0
150A	18	CUcCCGAUUcCACcAAUC	140–158	90.3
200A	13	CCaUCaUAAuCAC	194–206	89.6
285A	11	UGcUCuUCuAG	280–290	91.3
355A	16	UUcUCuCAUcCACuUU	352–370	88.0
415A	17	UUcGCCaUUGuUUGCUU	410–426	87.5
470A	13	CAuUCaGGUuGGA	466–478	93.0
600A	18	GUuCCaACUcCUUuCACC	591–608	87.6
615A	13	CUcCAuUGCuAUU	609–621	88.6
640A	17	CCaCGUuUGAuCAUUCU	628–644	86.7
760A	19	CCAuCAUuGCCcUCUgGGC	745–763	93.1
840A	12	CCcCUcAGAaUG	837–848	86.2
920A	13	CCuUCcCUUuCAA	917–929	87.2
1320A	21	GCUGCCAuAACGGUUGCUCUU	1308–1328	88.5
400A	11	CcAAAcUcUcC	398–405	88.6
727A	12	AuUuUCcUuUGA	722–732	91.6
950A	14	GAAuGGGuCUAuCC	943–957	90.6
2A	11	CAuUCuCAuUU	1146–1156	93.4
3A	16	CAuUCuCAuUUGAAGC	1141–1156	95.2
2B	11	UCcAGgGUaUU	1174–1184	88.2
Neg5*	11	CUGaACGCuUA	1262–1272	85.1
N*	17	AGACCUCUAUAGCAGCU	—	—

^a^Nucleotides in capital letter (A, C, G, U, D) are 2′-O-methyl-RNA nucleotides, lower case letters (a, c, g, u, d) are LNA nucleotides, ^b^numbering according to (+)RNA5 *-control ASO, Neg5 is complementary to double stranded region, N is not effective oligonucleotide used as control in published paper^17^. ^c^Calculated for type A influenza virus strains based on sequences database used in this study.

**Figure 6:**
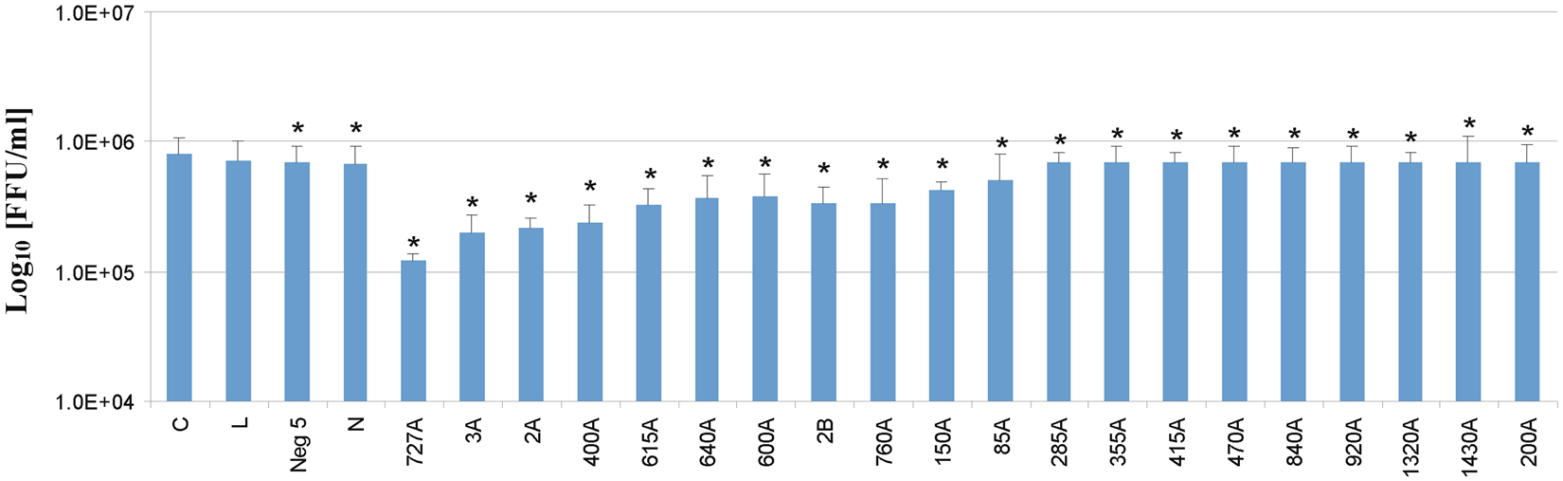
Antiviral activity of 750 nM ASOs in MDCK cells for A/California/04/2009 after 24 h postinfection. Antiviral activity was analyzed by immunofocus assay. The mean was calculated from three independent experiments, each containing three technical repeats and the standard deviation is shown. C - MDCK cells infected by virus, L - MDCK cells treated with Lipofectamine 2000 and infected, Neg 5 – MDCK cells transfected with control ASO Neg 5 and infected, N - MDCK cells transfected with control ASO N and infected. The remaining labels indicate MDCK cells transfected with indicated influenza A targeting ASOs and infected. Statistics for comparison to sample L were calculated using a two-tailed T-test (p < 0.01).

**Figure 7:**
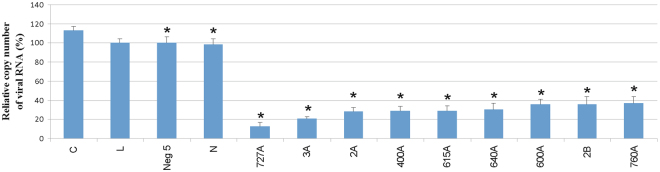
Antiviral activity of 750 nM ASOs in MDCK cells for A/California/04/2009 after 24 h postinfection. Antiviral activity was analyzed by qRT-PCR. The mean was calculated from three independent experiments each containing three technical repeats, each repeat was analyzed by three independent qRT-PCR reactions and the standard deviation is shown. C - MDCK cells infected by virus, L - MDCK cells treated with Lipofectamine 2000 and infected, Neg 5 – MDCK cells transfected with control ASO Neg 5 and infected, N - MDCK cells transfected with control ASO N and infected. The remaining labels indicate MDCK cells transfected with indicated influenza A targeting ASOs and infected. *Statistics for comparison to sample L were calculated using a two-tailed T-test (p < 0.01).

ASOs at a final concentration of 750 nM were tested against influenza virus proliferation in MDCK cells at 24 h post-infection using immunofluorescence assays and quantitative RT-PCR (qRT-PCR). 9 of 21 tested ASOs showed inhibition of influenza virus replication (Fig. 6). ASO 727A inhibited virus propagation more than seven-fold. Oligonucleotides targeting the previously described 121 nt motif in (+)RNA5 (2A, 3A, 2B) inhibited virus propagation 5.4, 5.0, 3.0 fold, respectively. These results correspond to a previous study with the same ASOs against single-cycle infectious influenza A virus (sciIAV) in a modified MDCK-HA cell line^8^. ASOs 2A, 3A and 2B inhibited influenza virus propagation in both assays, but the level of inhibition presented here was smaller, probably because experiments were conducted on a wild-type, fast replicating influenza virus in natural cells. Four-fold inhibition of viral replication was shown using ASO 400A. ASOs 615A, 760A, 640A and 600A inhibited virus propagation about three-fold. qRT-PCR confirmed the inhibitory potential of selected oligonucleotides. 87%, 79%, 72%, 71% 70%, 64% levels of inhibition were observed for 727A, 3A, 2A, 400A, 615A, 640A, respectively. 600A, 2B and 760A inhibited propagation of virus at the same level – 63% (Fig. 7).

### ASOs do not affect cell viability

Nine effective ASOs (727A, 2A, 3A, 2B, 400A, 615A, 760A, 640A, 600A) and two negative control oligonucleotides (Neg 5 and N) were tested for cytotoxicity (Figure S10). None of them caused differences in cell viability at concentration from 0.1 to 4 uM, suggesting that the inhibitory effect of the ASOs on influenza A/California/04/2009 H1N1 replication is not due to affecting cell viability, but is due to the specific targeting segment 5 (+)RNA.

## Discussion

The secondary structure of segment 5 (+)RNA based on experimental structure mapping, sequence/structure comparison and free energy minimization revealed structure motifs that are conserved across diverse influenza A strains. These motifs are thermodynamically stable and have high calculated base pair probability. Additionally, the discovered motifs persist at both low and high temperatures. All these results point to these stable and conserved motifs being functionally significant.

The effect of RNA folding on sequence evolution was most apparent in the UTRs. Elsewhere, however, there were specific pairs that showed greater-than-average MI (Supplementary Data 2). For example, the bp between nt 397–435, which occurs in the long hairpin formed between nt 388 and 444 (Fig. 1), has both higher-than-average MI and a high bp probability. Here, the high MI is possible because nt 397 is the first codon position for arginine, which, unlike most first codon positions, can vary from between two residues (A and C), but maintain the codon; nt 435 is the third codon position for threonine and can vary between any base. Thus, the increased flexibility of sequence evolution here, allows for the identification of pairing constraints, on top of the need to maintain codons. These results suggest that, while important, the coding constraints in segment 5 play a dominant role in sequence evolution. Indeed, a previous study of the suppression of synonymous codon use in segment 5 found only moderate suppression across the body of the sequence, but significant suppression toward the regions abutting the UTRs^7^.

The segment 5 (+)RNA construct comprises both cRNA and mRNA; thus the secondary structure studied *in vitro* highlights properties of both of these functional RNAs. Like all orthomyxoviruses, influenza A vRNA segments are transported from the cytoplasm into the nucleus, where they are used for replication^37^. Viral mRNAs, after being posttranscriptionally matured, are exported back to the cytoplasm for translation^38^. The processes of posttranscriptional maturation and export are both mediated by numerous RNA-protein associations—all of which may be affected by (pre)-mRNA structure. Once in the cytoplasm, additional posttranscriptional control mechanisms work to modulate gene expression. Many of these may also be influenced by RNA folding. The cRNA remains in the nucleus, where it is used to replicate additional vRNAs. In addition to the viral polymerase, cRNA also interacts with multiple copies of NP to form a ribonucleoprotein complex (cRNP). Cryo-electron microscopy, revealed that cRNP exhibits a circular, filamentous double-helical organization with defined termini, containing the viral RNA-dependent RNA polymerase at one end and a loop structure at the other end^39^. It was also noticed that sufficient flexibility exists within cRNP to form secondary RNA structures and favor suggestion of importance of cRNA secondary structure motifs. Recent work on NP associations in the RNP revealed that specific regions of RNA were dynamically interacting with protein^40^. Protein-free regions of cRNP can adopt structures that play roles and, vice versa, cRNA structure can affect NP binding dynamics: thus influencing replication rate and RNA stability, for example.

Our (+)RNA5 secondary structure model has circular folding that recapitulates the cRNA panhandle structure that is likely recognized by the viral polymerase, which distinguishes cRNA from vRNA. Interestingly, this interaction, as well as a downstream hairpin that occurs just before the NP protein start codon, were some of the best-characterized structures in our model: they had the highest mutual information scores of any base-paired regions (due to the lack of coding constraints) as well as high base pair conservation and partition function probability. The ability to identify these interactions *in silico*, based on probing data on “naked” RNA, lacking all interacting proteins, suggests that these motifs can form independently of the cRNP and form in advance of polymerase binding; this supports the idea that cRNA panhandle structure plays important function in the specificity of recognition by the viral polymerase. In addition, the novel hairpin identified nearby to the panhandle is also likely to play important roles, possibly in mRNA5, in the regulation of translation: e.g., the hairpin is directly upstream of the NP start codon, which is itself embedded within a long hairpin (Fig. 1).

Most revealed (+)RNA5 motifs remain the same, independent of the consideration of preferential local folding and also independent of the influence of the UTRs. Interestingly, only when local folding is constrained the structural motif at the 3′ end (nt 1478–1528) is formed (Supplementary Figure S7); this motif was predicted by two independent bioinformatics analyses and is highly conserved among influenza A strains^7,18^. It is possible that, preferentially local folding or circular folding motifs exist *in vivo* at certain times of the virus life cycle, and are promoted by viral or/and cellular factors. Hypothetically, changes of structure from circular to linear or linear to circular could take part in the life cycle of influenza virus; however, it likely only induces relatively small changes in global folding.

Local secondary structures of the UTRs and possible structurally labile regions revealed by temperature changes in (+)RNA5 could be functional, for example in mRNA5 (by influence on translation speed or interactions with translational factors) or in cRNA5 on replication (by different interactions with NP or in recognition and replication by the viral polymerase). Interestingly, a hairpin in region 577–593 is formed in both the UTR-deleted and temperature elevated models. This hairpin, with a 7-nt hairpin loop (CGGUGCA; Fig. 1), is shifted to have a 4-nt hairpin loop (CGGU; Supplementary Figure S5). This hairpin at nt 577–593 was also predicted by others^18^. It was noticed by these authors that a deletion of nt 583–585 occurs in an H1N1 strain isolated from Mongolia in 1991^18^. This deletion could influence the 4-nt hairpin loop (Supplementary Figure S5) by destabilizing it; this deletion will not, however, affect the 7 nt hairpin (Fig. 1), which may be an adaptation of certain strains. The sequence of nt 577–593 in the A/California/04/2009 H1N1 strain (GCCGCAGGUGCUGCGGU) also favors the alternative 7 nt hairpin.

A previous bioinformatics analysis showed a possible structured region for coding region 1–160 of (+)RNA5 (region 47–206 using entire (+)RNA5 numbering)^7^. Our experimental data, combined with new analyses, also proposed a secondary structure for this region. Interestingly, this region showed high base-pair conservation. Additionally, there are several compensating mutations for pairs: 5–56, 8–52, 24–38, 88–120, 93–116, 95–114, 64–180, 74–169, 93–116 and 95–114. Beside the (+)RNA panhandle structure, there are two hairpins: one just before the START codon and another that includes the START codon (Fig. 1). The two last nucleotides of the START codon are paired, making the closing base pairs of a long hairpin. Interestingly, for *Flaviviruses* it was showed that a small hairpin before a START codon influenced interactions with the ribosome, modulating viral mRNA translation rates^41^. The possible involvement of the (+)RNA5 START codon in a hairpin structure (nt 47–104) and preceding small hairpin (nt 16–39) could both potentially regulate translation in influenza.

Microarray mapping results show which regions from the full-length (+)RNA5 are accessible. Binding sites for microarray probes are in agreement with the proposed secondary structure model (Figs 1 and 5); however, not all single stranded regions are oligonucleotide binding sites. It is possible, that some regions could have additional tertiary interactions that inhibit binding (e.g. a 3D structured loop) or alternative intermolecular interactions. Such a pattern of oligonucleotide binding is typical for structured RNA^11,12,31,33^. The accessibility to oligonucleotide binding gives information on regions that are prone to intermolecular interactions, as well as regions that are mostly single stranded or weakly paired. This opens up the possibility for discovering new antiviral targets that can cause misfolding or block active regions of conserved motifs, which could then inhibit virus proliferation.

ASOs were designed on the basis of secondary structure of the entire segment 5 (+)RNA. ASOs (2A, 3A and 2B), that target a 121 nt conserved motif of (+)RNA5 and were previously tested on a modified, single-cycle infectious influenza A virus (sciIAV), inhibited the propagation of a wild influenza virus (Figs 6 and 7). Direct comparison showed that the level of inhibition was smaller for the wild type virus than for the previously studied sciIAV^8^. Better fitness of the wide type virus and/or different experimental conditions could explain this difference. Several new target regions were also revealed in our current study (Figs 6 and 7). The most effective ASO, 727A, targeted a long internal loop (nt 722–732). Another internal loop was bound by the effective ASOs 400A (398–405), 600A (591–608). Additionally, ASOs 615A, 640A targeting hairpin loops (609–621, 628–644, respectively) also decreased propagation of the virus. There was no correlation between the effectiveness of an ASO and its binding site sequence conservation (Table 2 and Figs 6 and 7). All sites were, on average, quite conserved. This suggests the most effective ASOs could have broad applications for targeting various influenza strains.

A region that overlapped (was one nt longer) the binding site of ASO 640A (628–645), was previously targeted using an antisense phosphorothioate-modified oligonucleotide (named NP628) specific to the influenza H5N1 virus (strain A/Tiger/Harbin/01/2002 H5N1)^17^. NP628 showed the best antiviral effect of three other phosphorothioate oligonucleotides tested by these authors. In addition, liposome mediated introduction of NP628 into mice partially protected them from lethal H5N1 virus challenge and resulted in a lower viral titer. The effectiveness of both oligonucleotides, 640A and NP628, are likely due to the accessibility of the (+)RNA secondary structure in this region.

Internal loops or hairpin loops were targeted by ASOs 760A (745–763), 2A (1146–1156) and 3A (1141–1156). Results for ASOs show that two domains are good targets for oligonucleotides – the previously described region formed by nt 1079–1194^8^ and the new region spanning nt 554–763. These two domains could take part in RNA-RNA or RNA protein interactions and be important in the influenza virus life cycle.

Oligonucleotide 760A partially targets the same region as published antisense oligonucleotide NP749 targeting strain A/Tiger/Harbin/01/2002^17^. NP749 reduced H5N1 virus titer and NP mRNA level. Both results show that targeting this region of different influenza strains is effective and also that this region is functionally important. We could not compare directly the antiviral effect because different conditions and method of analysis used. It is, however, notable that conserved regions for different strains of H5N1 influenza are good targets for antisense oligonucleotides and their effectiveness is correlated to the revealed RNA secondary structure described here. This model will facilitate future research into the functional analysis of influenza RNA as well as its therapeutic targeting.

## Methods

### Plasmid

Plasmid pPol1 contains sequence of segment 5 of influenza virus strain A/Vietnam/1203/2004(H5N1) was received from Professor Beak Kim (University of Rochester).

### Oligonucleotides synthesis and labeling

Primers for PCR, qRT-PCR and reverse transcription, DNA oligonucleotides for RNase H cleavage, ASOs (Table 2, Supplementary Tables S1–S3) were synthesized by the phosphoramidite approach on MerMade synthesizer. Primers were deprotected and purified according to published protocols^42,43^. Concentrations of all oligonucleotides were measured using a Spectrophotometer UV (NanoDrop2000 Thermo Scientific). Primers for reverse transcription were synthesized with aminolinker on 5′ end and after deprotection and desalting were labeled from the 5′ end with fluorophores: 5-FAM, 5-ROX, 6-TAMRA, and 6-JOE (dyes from ANASPEC). For labeling the reaction mixture containing 300 µg primer (11 µl in H_2_O) and 220 µg one of diluted in DMSO fluorophores (14 µl) and 75 µl 0.1 M sodium tetraborate, pH 8.5 was incubated overnight in room temperature in shaker oscillating at low speed. Labelled primers were precipitated with ethanol and purified by electrophoresis on 12% denaturing PAGE.

### RNA synthesis

DNA template for *in vitro* transcriptions of (+)RNA5 and (+)RNA5-ORF were obtained by PCR from vector pPol1 using primers For_c5, Rev_c5 and FM5, RM5, respectively (Supplementary Table S1). DNA was purified using PCR/DNA Clean-up purification Kit (Eurx). *In vitro* transcription reaction was performed using an Ampliscribe T7 Flash kit (Epicenter) according to manufacture protocol. Product was purified using RNeasy MiniElute Cleanup Kit (Qiagen). Integrity and purity of samples were checked on agarose gel.

### RNA folding

RNA ((+)RNA5 and (+)RNA5-ORF) in folding buffer (100 mM KCl and 5 mM MgCl_2_, 50 mM HEPES pH 7.0 or 300 mM KCl, 5 mM MgCl_2_, 50 mM HEPES pH 7.0) was heated in 65 °C for 5 min. and slowly cooled to room temperature. Folding was analyzed by native gel electrophoresis using 0.8% agarose gel running at 4 °C with low voltage. Under these conditions, one band was observed (Supplementary Figure S1). For chemical mapping, the above 100 mM KCl buffer was used and for microarrays mapping and RNase H cleavage the higher salt buffer with 300 mM KCl was used.

### Chemical mapping

Prior to chemical mapping RNA was folded as was described above. Chemical mapping was carried out with 6 mM DMS (dimethyl sulfate), 16.5 mM CMCT (1-cyclohexyl-(2-morpholinoethyl) carbodiimide metho-p-toluene sulfonate), 6.7 mM kethoxal or 1 mM NMIA (N-methylisatoic anhydride) for 15, 20, 30 or 40 min, respectively, at selected temperature (23 °C or 37 °C). Control reaction were performed in the same conditions but without mapping reagents. Modified nucleotides were read out by primer extension using a stechiometry of 2 pmol primer/1.5 pmol RNA. Primer extension was performed at 55 °C with reverse transcriptase SuperScript III (Invitrogen) using manufacturer’s protocol. Primers labeled by 6-JOE were used for detection of modification by DMS, CMCT, NMIA or kethoxal. Primers labeled by 5-FAM were used for detection of reverse transcription products that serve as control of quality of RNA. For each primers labeled with 5-ROX and 6-TAMRA there were prepared ddNTP ladders: (most often ddGTP and ddATP). cDNA fragments were separated by capillary electrophoresis (DNA Research Center, Poznan).

### Processing of chemical mapping data

Data were analyzed with the ShapeFinder program^44^. Quantitative NMIA reactivities for individual datasets were normalized to a scale in which 0 indicates an unreactive site and the average intensity at highly reactive sites is set to 1.0. The normalization factor for each datasets was determined by first excluding the most reactive 2% of peak intensities and then calculating the average for the next 8% of peak intensities. All reactivities were then divided by this average. In this scale, reactivities ≥0.700 are considered as strong, 0.500–0.700 as medium and <0.500 as weak, but all calculated reactivities were used for prediction of secondary structure. Nucleotides with no data were indicated as −999. Normalized SHAPE reactivities from each primer extension reaction were processed independently. DMS, CMCT, kethoxal modifications analysis was equivalent to that described above, but only strong and medium modification were used in prediction. For all SHAPE and DMS, CMCT, kethoxal mapping analysis at least three datasets were obtained from each primer and the average of results was used in the RNAstructure 5.5^45,46^ for prediction of secondary structure.

### Prediction of secondary structure using chemical mapping results

For determination of RNA secondary structure RNAstructure5.5 program was used with incorporated experimental data. RNAstructure^45^ uses thermodynamic parameters^46,47^ and free energy minimization for prediction of base pairing^48^. SHAPE reactivities were converted to pseudo free-energy change terms to restrain predictions. For this purpose the text file with normalized SHAPE reactivity (as described above) was input to RNAstructure 5.5 using “Read SHAPE reactivity—pseudo free energy” mode with slope 1.6 and intercept −0.6 kcal/mol^49^. DMS, CMCT, and kethoxal mapping data were also introduced at the same time, using “chemical modification” mode to apply only strong DMS, CMCT and kethoxal modifications as constraints^50,51^.

### Conservation analysis of model structure

All available influenza A strains were downloaded from the NCBI influenza virus resource^29^, only non-redundant, full-length sequences were taken. Genome alignment of influenza sequences was performed using MAFFT^52^ using the FFT-NS-1 fast alignment approach. Base pairing conservation was analyzed by counting model pair nucleotides in the alignment using a Perl script (Supplementary File). The base pair probabilities were calculated using the program RNAstructure^45^ and the mutual information (MI) score was calculated using a module within the alignment editor BioEdit^53^.

### RNase H assays

For RNase H assays RNA was folded as described above. Then, 3 pmol of DNA oligonucleotide in buffer with DTT (final concentration 1 mM) and 5 units of RNase H were added to final volume 20 μl. A control reaction was prepared equivalently but without DNA oligomer. Reactions were incubated for 30 min at 37 °C and then RNase H was inactivated by incubation for 10 min at 65 °C. Samples were precipitated with ethanol and cleavage sites were identified with primer extension using stoichiometry 2 pmol primer/1.5 pmol RNA. Primers labeled by JOE were used for detection of RNA cleavage. Primers labeled by FAM were used for control reactions. For each primers labeled with ROX and TAMRA there were prepared ddNTP ladders: most often ddGTP and ddATP.

### Hybridization to Isoenergetic Microarrays

Isoenergetic microarrays were prepared and microarrays mapping was performed similarly to published procedures^31,54^. Microarray probes were complementary to (+)RNA5 step-by-step giving that whole studied RNA was scanned (Supplementary Data 3). Probes were 2′-O-methyl oligonucleotide pentamers and hexamers with incorporated LNA nucleotides and 2,6-diaminopurine riboside (LNA or 2′-O-methylated nucleotide)^32,34^. Probes were spotted in duplicate. Spotting buffer, monomer U, and pentamer UUUUU (2′-O-methyl-RNA), which should show no binding to RNA, were also printed on the microarray as internal negative controls. Silanized slides were coated with 2% agarose activated by NaIO4 and microarrays were printed in the European Center of Bioinformatics and Genomics in Poznan, Poland with NanoPrint microarray printer (Arrayit). Printed microarrays were incubated for 12 h at 37 °C in 50% humidity. The remaining aldehyde groups on microarrays were reduced with 35 mM NaBH_4_ solution in phosphate-buffered saline solution and ethanol (3:1 v/v). Then slides were next washed (3x) in water at room temperature, next in 1% SDS solution at 55 °C (1x), and finally in water at room temperature (3x) and dried at room temperature.

About 200,000 cpm of 5′ radioactive labeled RNA (ca. 10 nM) was folded as described above before hybridization to isoenergetic microarrays. Hybridizations were carried out in folding buffer for 18 h at 23 or 37 °C in 100% humidity. Microarrays were washed for 3 min at the same temperature and buffer composition as hybridization occurred and then dried by centrifugation. Hybridization was visualized by exposure to a phosphorimager screen and quantitative analysis was performed with ArrayGaugeV2.1. Binding was considered strong, medium and weak, when the integrated spot intensity was ≥1/3, ≥1/9, or ≥1/27 of the strongest integrated spot intensity, respectively. The average of at least three repeats is presented. Alternative binding sites were predicted using RNA/RNA thermodynamics.

### Cells and viruses

Madin-Darby canine kidney (MDCK) cells (Sigma) were propagated in Dulbecco’s modified Eagle’s medium (DMEM) supplemented with 10% fetal bovine serum (FBS) and 1% PSG (penicillin—100 U/ml, streptomycin—100 mg/ml, l-glutamine—2 mM) at 37 °C with 5% CO_2_. Influenza A/California/04/2009 (H1N1) titers were determined with standard plaque assays^55,56^. A/California/04/2009 (H1N1) virus was a gift from Prof. Luis Martinez-Sobrido, University of Rochester.

### Antiviral test of ASOs in MDCK cells

One day before transfection, 2.5 × 10^6^ cells were seeded in a 100-mm dish. Before each transfection, Lipofectamine 2000 (Thermo Fisher Scientific) was diluted with the Opti-MEM I Reduced Serum Medium (Opti-MEM) and incubated 10 min at RT. Then 15 µl of this solution was mixed gently with 15 µl ASO in OptiMEM and incubated 30 min at room temperature. To each of prepared ASO solution, 300 µl of 7.5 × 10^4^ MDCK cells in media was added. Next, the mixture was divided for 100 µl and added to three wells in a 96-well plate. Final concentration of ASO was 750 nM. Cells were incubated at 37 °C in air with 5% CO_2_ and after 12 h medium was changed for fresh one followed by incubation of another 6 h. Transfection efficiency was checked on flow cytometer and optimized using fluorescently labeled (TAMRA) oligonucleotide. Several concentrations were tested: 4 µM, 2 µM, 1 µM, 0.75 µM, 0.5 µM, 0.25 µM and 0.1 µM. The efficiency of 750 nM was the highest and similar as for 1–4 µM concentration and therefore was chosen for further experiments. In each experiment, transfection was checked using labeled NEG5 (ROX) under fluorescence microscope.

After 18 h posttransfection with ASO, 0.01 MOI (multiplicity of infection) of A/California/04/2009 (H1N1) was used to infect MDCK cells. Infection medium consist of PBS supplemented with 0.3% bovine albumin (BA) and 1% PS (penicillin-100 U/ml, streptomycin-100 mg/ml). After 1 h incubation at RT on a rocking platform, the infection medium was changed for the postinfection medium containing DMEM supplemented with 0.3% BA, 1% PSG, and 1 mg/ml tosyl–sulfonyl phenylalanyl chloromethyl ketone (TPCK)-treated trypsin (Sigma). Infected MDCK cells were next incubated at 33 °C in air with 5% CO_2_ for 24 h. Next, cell culture supernatants (CCS) were collected. Viral titers were analyzed with the immunofocus assay and vRNA amount is measured using qRT-PCR.

### Immunofocus assay

Serial dilution of cell culture supernatants were used to infect MDCK cells cultured on 96-well plates. Infection was conducted for 1 h at RT. After incubation, the infection medium was changed for the postinfection medium and infected cells were maintained at 33 °C in air with 5% CO_2_ for 8–10 h. Next, 4% paraformaldehyde and 0.5% Triton X-100 in PBS were used to fixed and then permeabilize cells. Cells were incubated with a blocking buffer containing 3% bovine serum albumin in PBS for 1 h. After blocking, the solution was replaced with mouse anti-influenza A monoclonal primary antibody targeting NP protein (MAB8257 Merck Merc Millipore) diluted in the blocking buffer (1 µg/ml) and incubated for 1 h at 37 °C. FITC-conjugated secondary rabbit anti-mouse IgG antibody (AP160F Merck Millipore) diluted in blocking buffer (1:150) was used to detect primary antibody by incubation 1 h at 37 °C. Fluorescent-forming units (FFU/ml) were counted under a fluorescent microscope. The mean titer from triplicates and the standard deviation were calculated with Microsoft Excel software.

### Extraction of total RNA from cells

For real-time PCR cells were seeded on 24-well plate. Transfection and infection were conducted as describe above (proportional to 24-well plates format). Following the removal of supernatant, infected cells were washed with PBS and total RNA was extracted from cells using Chomczynski and Sacchi method^57^ with TRI Reagent (Sigma-Aldrich). RNA quality was checked on agarose gel.

### Viral RNA reference standards for qRT-PCR

Viral RNA of segment 7 (vRNA7) was used as a reference standard for the quantitation procedure^58^. To obtain vRNA7 standard first total RNA was extracted from infected MDCK cells. Reverse transcription was carried out using Superscript III reverse transcriptase (Invitrogen) and primer RT (Supplementary Table S3) which is complementary to the 22 nucleotides of the 3′ end of vRNA7. Obtained cDNA was used as a template for PCR reaction with primers carrying T7 RNA polymerase promoter sequence. PCR products were *in vitro* transcribed using the AmpliScribe T7-Flash Kit (Epicentre Biotechnologies), following manufacturer’s protocol. The RNA transcript was purified using the RNeasy MinElute Spin Columns (Qiagen). Quality of RNA sample was verified by electrophoresis on agarose gel and concentration was determined by spectrophotometry. 10-fold serial dilutions of RNA were prepared and reverse transcription with RT primer was carried out. cDNA was then amplified in qPCR reaction to obtain standard curve^59^.

### qRT-PCR

For cDNA preparation, a separate reverse transcription step was performed using a SuperScript III (Invitrogen) according to manufacture protocol. 500 ng of total cellular RNA serve as temple for reaction and 2 uM of RT primer (Supplementary Table S3) was used. The qPCR step was performed using 5x HOT FIREPol® Probe qPCR Mix: (Cytogen) on a C1000 Touch^TM^ Thermal Cycler (BioRad) using software version v.3.1.1517.0823 in 96-well format. qPCR reaction components were set-up in triplicate according to the manufacturer’s instruction. 1ul cDNA from the RT step and 10 pmol of QF, QR and 10 pmol Taqman probe Q (Supplementary Table S3) were added to reaction Mix to the total volume of 10 ul. Primers were specific to a highly conserved region of the vRNA7. Standard cycling conditions were 95 °C for 15 min, followed by 49 cycles of 95 °C for 20 s, 60 °C for 60 s. A negative water sample was included in each run. The quantity of viral RNA molecules was determined using the vRNA7 standard curve, processed in parallel.

### Cytotoxicity assays

MDCK cells were transfected with ASOs in concentration from 1 uM to 4 uM and seeded on 96-well plate as for experiments of antiviral tests described above. After transfection cells were incubated at 37 °C in air with 5% CO_2_ and after 12 h medium was changed for fresh one followed by incubation of another 6 h. MDCK cells treated with LPF-2000 were also evaluated. The cytotoxicity of ASOs were evaluated using 1% 3-(4,5-dimethylthiazol-2-yl)-2,5-diphenyltetrazolium bromide (tetrazolium). MDCK cells were incubated for 2 h with tetrazolium at 37 °C in air with 5% CO_2_ and after 2 h, solution was changed for DMSO (100 ul/well) and incubated for 15 min. in dark. The rate of formazan formation was determined by measuring the absorbance (570–650 nm) on xMark^TM^ Microplate Spectrophotometer (BioRad). Cells enzymes are capable of reducing the tetrazolium to its insoluble formazan. The 570–650 nm reading value is directly proportional to the number of living cells. Cytotoxicity assays were normalized to viability of cells treated only with Lipofectamine 2000. The mean and standard deviation were calculated with Microsoft Excel software from three independent experiments each containing three technical repeats.

## Electronic supplementary material

Supplementary Information

Supplementary Data 1

Supplementary Data 2

Supplementary Data 3

Supplementary File
