# Supplementary material for: Influenza virus segment 5 (+)RNA - secondary structure and new targets for antiviral strategies
Source: Sci Rep. 2017 Nov 8;7:15041. doi: 10.1038/s41598-017-15317-5 (PMC5678188; doi:10.1038/s41598-017-15317-5)
Supplement: Supplementary file 1 — Supplementary Information [file 41598_2017_15317_MOESM1_ESM.pdf]

Supplementary Information

Influenza virus segment 5 (+)RNA - secondary structure and new targets for antiviral strategies

Marta Soszynska-Jozwiak[1], Paula Michalak[1], Walter N. Moss[2], Ryszard Kierzek[1], Julita Kesy[1], Elzbieta Kierzek[1]*

[1]Institute of Bioorganic Chemistry Polish Academy of Sciences, 61-704 Poznan, Noskowskiego 12/14, Poland

[2]Roy J. Carver Department of Biophysics, Biochemistry and Molecular Biology, Iowa State University, Ames, IA 50011, United States of America

*Corresponding author: E-mail: elzbieta.kierzek@ibch.poznan.pl, Institute of Bioorganic Chemistry Polish Academy of Sciences, 61-704 Poznan, Noskowskiego 12/14, Poland, Tel.: +4861-853-8503; Fax: +4861-852-0532.

Name	Length (nt)	Sequence 5’→3’ [a]
For_c5	45	GCGTAATACGACTCACTATAGGGAGCAAAAGCAGGGTAGATAATC
Rev_c5	22	AGTAGAAACAAGGGTATTTTTC
FM5	40	GCGTAATACGACTCACTATAGGGATGGCGTCTCAAGGCAC
RM5	29	TTAATTGTCATACTCCTCTGCATTGTCTC

Table S1. Primers for polymerase chain reaction using to obtain DNA template for (+)RNA5 and coding region 5. [a] The underlined nucleotides residues are the polymerase T7 promoter.

Name	Length (nt)	Complementary region in segment 5 RNA	Sequence 5’→3’ [a]
M1[a]	32	1533-1565	AGTAGAAACAAGGGTATTTTTCTTTAATTGTC
RTM5[a]	27	1516-1540	TTAATTGTCATACTCCTCTGCATTGTC
M2[a]	24	1302-1324	CCATAATGGTCGCTCTTTCGAAG
M3[a]	20	1047-1066	GGTCCTCAAATGCTGCAGAG
M4[a]	24	782-805	CAATTTCAGCATTCCCAGGATTTC
M5[a]	20	517-536	CACATCCTTGGGTCCATTCC
M6[a]	25	259-283	CCAGGTATCTGTTCCTTCTTTCATC

Table S2. Primers for reverse transcription. [a] Primers were labeled with 6-TAMRA, 5-FAM, 6-JOE or 5-ROX at 5’ end.

Name	Length (nt)	Sequence 5’→3’
RT	22	ATGAGTCTTCTAACCGAGGTCG
PF[a]	52	GCGTAATACGACTCACTATAGGGTTACTCTAGCTCTATGTTGACAAAATGAC
PR	26	ATGAGTCTTCTAACCGAGGTCGAAAC
QF	23	AGACCAATCTTGTCACCTCTGAC
QR	26	AGGGCATTTTGGACAAAGCGTCTACG
Q[b]	27	TCACCGTGCCCAGTGAGCGAGGACTGC

Table S3. Primers for qRT-PCR. [a] The underlined nucleotides residues are the polymerase T7 promoter. [b] Primer was labeled with, 5-FAM at 5’ end 6-TAMRA at 3’ end.

Binding site[a]	Probe sequence[b]	Strength of probe binding[c]	ΔG°_{37} of duplex for complementary binding site[d] (kcal/mol)	Nucleotide of RNA target complementary to 3'G of hexamer probe	ΔG°_{37} of duplex for possible mismatched sites (kcal/mol)[e]
11/ 122/ 1422	CCcUg	S	-5.5 (-9.22) 11<br>-5.4 (-9.22) 122<br>-6.2 (-9.22) 1422	-	-
12 /551 /929	AcCcUg	M	-9.3 (-12.12) 12<br>-7.0 (-10.18) 551<br>-7.6 (-10.18) 929	12C<br>551A<br>929A	-8.6 (1422)<br>-7.9 (122)
95	CcCcA	M	-8.7 (-9.88) 95	-	-5.7 (154)
96/ 1429	UCcCc	M	-8.8 (-9.54) 1429<br>-8.6 (-9.54) 96	-	-
250/ 540/ 936	dGdGdg	M	-7.7 (-12.84) 250<br>-7.7 (-12.84) 936<br>-7.4 (-12.84) 540	250C<br>936C<br>540C	-
301 (350)	uCcCg	S	-7.8 (-9.61) 301<br>-8.1 (-9.61) 350	-	-6.9 (155/156)<br>-6.9 (96/97)<br>-6.9 (728/729)<br>-6.9 (791/792)<br>-6.9 (206/207)<br>-6.9 (1429/1430)<br>-6.7 (644/645)<br>-6.7 (1091/1092)<br>-6.6 (605/606)<br>-6.0 (95/96)
630	AuCcGg	S	-6.8 (-8.65) 630	630G	-5.0 (349/350)<br>-4.8 (300/301)
953/ 1479	dDgGdg	M	-4.5 (-10.59) 953<br>-4.7 (-10.59) 1479	953A<br>1479U	-
954/ 1480	dDdGgg	S	-4.8 (-7.93) 1480<br>-4.2 (-7.93) 954	1480U<br>954U	-
956	gGdDdg	M	-7.5 (-11.68) 956	956C	-4.2 (1477/1478)
1299/ 1477	GgDdGg	M	-9.3 (-12.75) 1477<br>-9.2 (-12.75) 1299	1477C<br>1299C	-7.2 (955/956)
1431/ 1529	DcUcCg	M	-7.2 (-10.04) 1431<br>-7.8 (-10.04)1529	1431G<br>1529A	-7.0 (156/157)<br>-7.0 (207/208)<br>-6.5 (388/389)<br>-6.5 (666/667)<br>-6.5 (422/423)<br>-6.5 (567/568)<br>-5.6 (95/96)
1555	CDdGgg	M	-8.7 (-12.32) 1555	1555C	-6.1 (1480/1481)
154/ 1202	CcCdA	M	-6.4 (-8.23) 154<br>-5.7 (-8.23) 1202	-	-6.2 (94/95)<br>-6.2 (790/791)<br>-5.5 (356/357)
155/ 791	uCcCa	S	-7.0 (-8.86) 155	-	-6.9 (206/207)<br>-6.9 (301/302)<br>-6.9 (728/729)<br>-6.9 (1429/1430)<br>-6.9 (350/351)<br>-6.9 (791)<br>-6.9 (96/97)<br>-6.7 (644/645)

					-6.6 (1091/1092)<br>-6.6 (605/606)<br>-4.7 (1202/1203)
156/ 207/<br>1430	CUcCc	S	-8.5 (-9.16) 156<br>-8.3 (-9.16) 207<br>-7.6 (-9.16) 1430	-	-5.9 (1514/1515)<br>-5.8 (388/389)<br>-5.8 (567/568)<br>-5.7 (301/302)<br>-5.6 (666/667)<br>-5.6 (728/729)<br>-5.6 (422/423)<br>-5.4 (644/645)<br>-5.4 (96/97)
264	UcUuUcA	M	-6.8 (-9.12) 264	-	-6.9 (705)<br>-6.8 (921)<br>-6.7 (237/238)<br>-6.7 (775/776)<br>-6.4 (344/345)<br>-5.8 (845)<br>-5.8 (424/425)<br>-5.8 1224/1225)<br>-5.7 (643/6440<br>-5.6 (1090/1091)
13/ 601/ 930	UdCcCg	M	-8.0 (-9.85) 601<br>-8.0 -9.85) 13<br>-7.7 (9.85) 930	13A<br>601G<br>930A	-6.4 (551/552)<br>-6.2 (300/301)
365/ 501/ 753/<br>778/ 1311	GcUcUg	M	-7.8 (-10.26) 365<br>-7.8 (-10.26) 501<br>-8.2 (-10.26) 753<br>-8.3 (-10.26) 778<br>-8.2 (-10.26) 1311	365G<br>501G<br>753A<br>778G<br>1311A	-8.9 (122/123)<br>-6.6 (12)
237/ 344/ 362/<br>364/ 424/ 500/<br>668 /705/ 775/<br>777/ 845/ 921/<br>923	CUcUcg	S	-8.6 (-11.51) 775<br>-8.2 (-11.51) 500<br>-7.9 (-9.08) 424<br>-7.9 (-9.08) 668<br>-7.9 (-9.08) 845<br>-7.7 (-9.08) 923<br>-7.7 (-9.08) 344<br>-7.7 (-9.08) 237<br>-7.7 (-9.08) 705<br>-7.1 (-9.08) 921<br>-7.1 (-9.08) 777<br>-7.7 (-9.08) 362<br>-7.7 (-9.08) 364	775C<br>500C<br>424G<br>668G<br>845U<br>923A<br>344G<br>237A<br>705U<br>921U<br>777A<br>362U<br>364A	-7.0 (156)<br>-7.0 (207)<br>-6.3 (1430)
343/ 389/ 423/<br>568/ 667/<br>1515	UcUcCg	S	-8.1 (-9.81) 343<br>-8.1 (-9.81) 423<br>-7.9 (-9.81) 389<br>-7.9 (-9.81) 568<br>-8.1 (-9.81) 667<br>-8.6 (-11.89) 1515	343A<br>423U<br>389A<br>568A<br>667U<br>1515C	-8.1 (1428/1429)<br>-7.2 (96)<br>-6.1 (1528/1529)<br>-5.2 (775/776)
386/ 425/ 669/<br>846/ 1089/<br>1110/ 1128/<br>1243/ 1526	CcUcUg	M	-9.5 (-12.03) 1128<br>-9.4 (-12.03) 1243<br>-9.3 (-12.03) 1089<br>-9.2 (-12.03) 1526<br>-7.6 (-10.09) 846<br>-7.6 (-10.09) 425<br>-7.5 (-10.09) 386<br>-7.5 (-10.09) 1110<br>-6.9 (-10.09) 669	1128C<br>1243C<br>1089C<br>1526C<br>846G<br>425G<br>386A<br>1110A<br>669G	-7.7 (1422/1423)
341/ 387/ 398/<br>426/, 566/	uCcUcg	M	-9.1 (-12.42) 566<br>-8.1 (-9.99) 341	566C<br>341G	-8.1 (1428/1429)<br>-7.2 (96)

847/ 1062/,<br>1111/ 1225/<br>1367/ 1527			-8.1 (-9.99) 387<br>-8.1 (-9.99) 1527<br>-8.1 (-9.99) 1225<br>-7.9 (-9.99) 847<br>-7.9 (-9.99) 398<br>-7.8 (-9.99) 1367<br>-7.8 (-9.99) 1062<br>-7.8 (-9.99) 426<br>-7.8 (-9.99) 1111	387A<br>1527A<br>1225A<br>847A<br>398G<br>1367U<br>1062U<br>426A<br>1111A	
206/ 605/ 644/<br>728/ 1091	UcCcUg	S	-7.9 (-10.47) 728<br>-7.9 (-10.47) 206<br>-7.8 (-10.47) 644<br>-7.7 (-10.47) 1091<br>-7.7 (-10.47) 605	728A<br>206A<br>644G<br>1091G<br>605U	-8.9 (1428/1429)<br>-8.5 (1422/143)<br>-7.6 (11/12)<br>-7.6 (96)<br>-7.3 (122)<br>-5.9 (928/929)<br>-5.9 (550/551)
645	AuCcCg	S	-7.3 (-9.11) 645	645A	-8.1 (350)<br>-7.8 (301)<br>-6.9 (155/156)<br>-6.9 (728/729)<br>-6.9 (791/792)<br>-6.9 (206/207)<br>-6.9 (96/97)<br>-6.7 (1091/1092)<br>-6.7 (605/606)
880/ 1556	DcDdGg	S	-6.4 (-11.31) 1556<br>-4.8 (-8.73) 880	1556C<br>880G	-

Table S4. Isoenergetic microarrays probes that bind strongly and moderately to (+)RNA5 and their thermodynamic properties. a - probable binding sites of probes, sites are denoted by the middle nucleotide of the complementary RNA region (or two nucleotides for probes with an even number of nucleotides), underlined are sites accessible according to (+)RNA5 secondary structure (Figure 1); b - nucleotides in capital letter (A, C, G, U, D) are 2'-O-methyl-RNA nucleotides, lower case letters (a, c, g, u, d) are LNA nucleotides, D and d are 2,6 – diaminopurine (2'-O-methyl type or LNA, respectively); c - binding was considered strong (S), medium (M) and weak (W), when the integrated intensities were ≥1/3, ≥ 1/9 and ≥ 1/27 of the strongest intensity. Hybridization condition: buffer 300 mM KCl, 5 mM $MgCl_2$, 50 mM HEPES, pH 7.0, 37°C; d - $\Delta G°_{37}$ calculated as RNA/RNA duplex and, in parenthesis, as modified probe/RNA duplex for listed binding sites [1,2]; e - calculated in RNAstructure program as RNA/RNA duplex and, in parenthesis, the site of binding for which calculation was done. Only alternative binding sites of probes with thermodynamic stability more favorable than -4.0 kcal/mol are noted.

Sites of RNase H cleavage	Sequence of DNA oligonucleotide	Complementary region for DNA in (+)RNA5
157^{s}	CTCCCAATG	150-158
423^{s}, 847^{s}, 923s, 925^{s}	CTCTCCATT	418-426
$604\text{-}605^{s}$	CTACCCC	598-604
$262\text{-}264^{s}$	CTTTCATCA	258-266
287^{s}	GTTCTTCCA	281-289
365^{s}	CTCTCTCAC	358-365
760^{s}	CCATCATTG	755-763
356^{s}	CCCATTTCC	350-358
620^{s}	GCTCCATC	615-622
642^{s}	CTCGTTTTA	635-643
1153^{s*}, 1154^{s*}	TGTTCTCA	1149-1156
$58\text{-}60^{s}$, $997\text{-}999^{s}$, 729^{w}	TTTCCCTTT	724-732
880^{w}	CACAAGCAG	875-883
473^{w}	ATTTAGATTG	468-477
1181^{w}	TCAAGAGTG	1176-1184
705^{w}	ATTCTCTCA	703-710
-	CAAATCCTC	396-404
-	AAGGTCCTC	1060-1068
-	GGATCTATT	943-953

Table S5. RNase H cleavage sites induced by DNA oligonucleotides in (+)RNA5. s - sites of strong RNase H cleavage, w - sites of weak RNase H cleavage ”-„ no cleavage. *-published data [3].

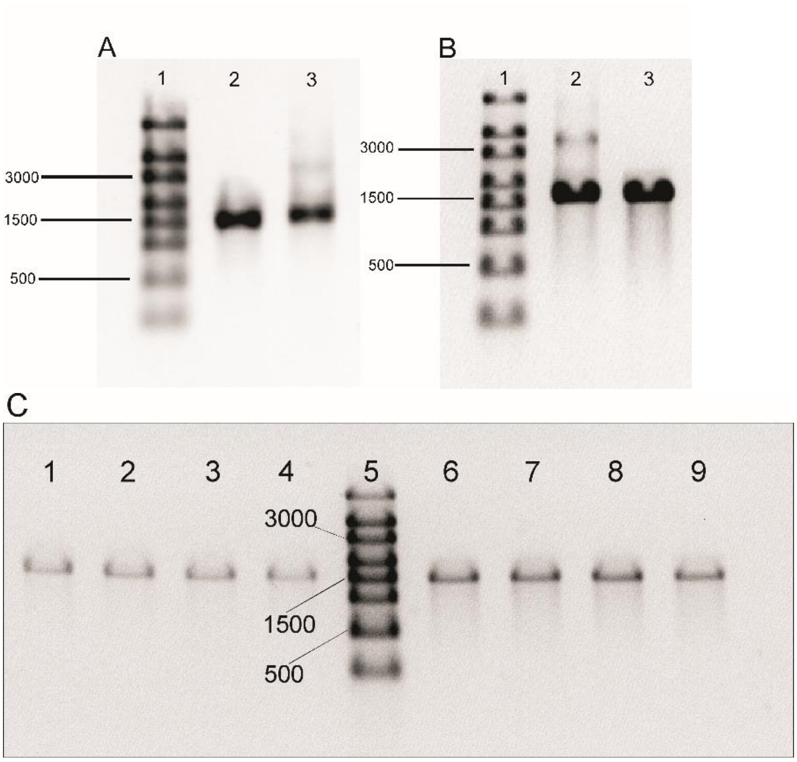

Figure S1. A) - (+)RNA5 analysis by agarose gel electrophoresis. 1 - RNA Ladder - RiboRuler High Range RNA Ladder; 2 - (+)RNA5 after folding described in Methods (in buffer: 100 mM KCl and 5 mM $MgCl_2$, 50 mM HEPES pH 7.0) and slow cooling to 23°C; 3 - (+)RNA without folding – the homodimer is present. B) - (+)RNA5-ORF analysis by agarose gel electrophoresis. 1 - RNA ladder (RiboRuler High Range RNA Ladder); 2 - (+)RNA5-ORF without folding – the homodimer is present; 3 - (+)RNA5-ORF after folding described in Methods (in buffer 100 mM KCl and 5 mM $MgCl_2$, 50 mM HEPES pH 7.0) and slow cooling to 23°C. C) - (+)RNA5 after folding described in Methods in appropriate buffer: 1 - (+)RNA5 after folding in buffer: 100 mM KCl, 5 mM $MgCl_2$, 50 mM HEPES pH 7.0 and slow cooling to 23°C; 2 - (+)RNA5 after folding in buffer: 100 mM KCl and 5 mM $MgCl_2$, 50 mM HEPES pH 7.0 and slow cooling to 37°C; 3 - RNA5 after folding in buffer: 300 mM KCl, 5 mM $MgCl_2$, 50 mM HEPES pH 7.0 and slow cooling to 23°C; 4 - (+)RNA5 after folding in buffer: 300 mM KCl, 5 mM $MgCl_2$, 50 mM HEPES pH 7.0 and slow cooling to 37°C; 5 - RNA Ladder - RiboRuler High Range RNA Ladder; 6 - (+)RNA5-ORF after folding in buffer: 100 mM KCl, 5 mM $MgCl_2$, 50 mM HEPES pH 7.0 and slow cooling to 23°C; 7 - (+)RNA5-ORF after folding in buffer: 100 mM KCl, 5 mM $MgCl_2$, 50 mM HEPES pH 7.0 and slow cooling to 37°C; 8 - (+)RNA5-ORF after folding in buffer: 300 mM KCl, 5 mM $MgCl_2$, 50 mM HEPES pH 7.0 and slow cooling to 23°C; 9 - (+)RNA5-ORF after folding in buffer: 300 mM KCl and 5 mM $MgCl_2$, 50 mM HEPES pH 7.0 and slow cooling to 37°C.

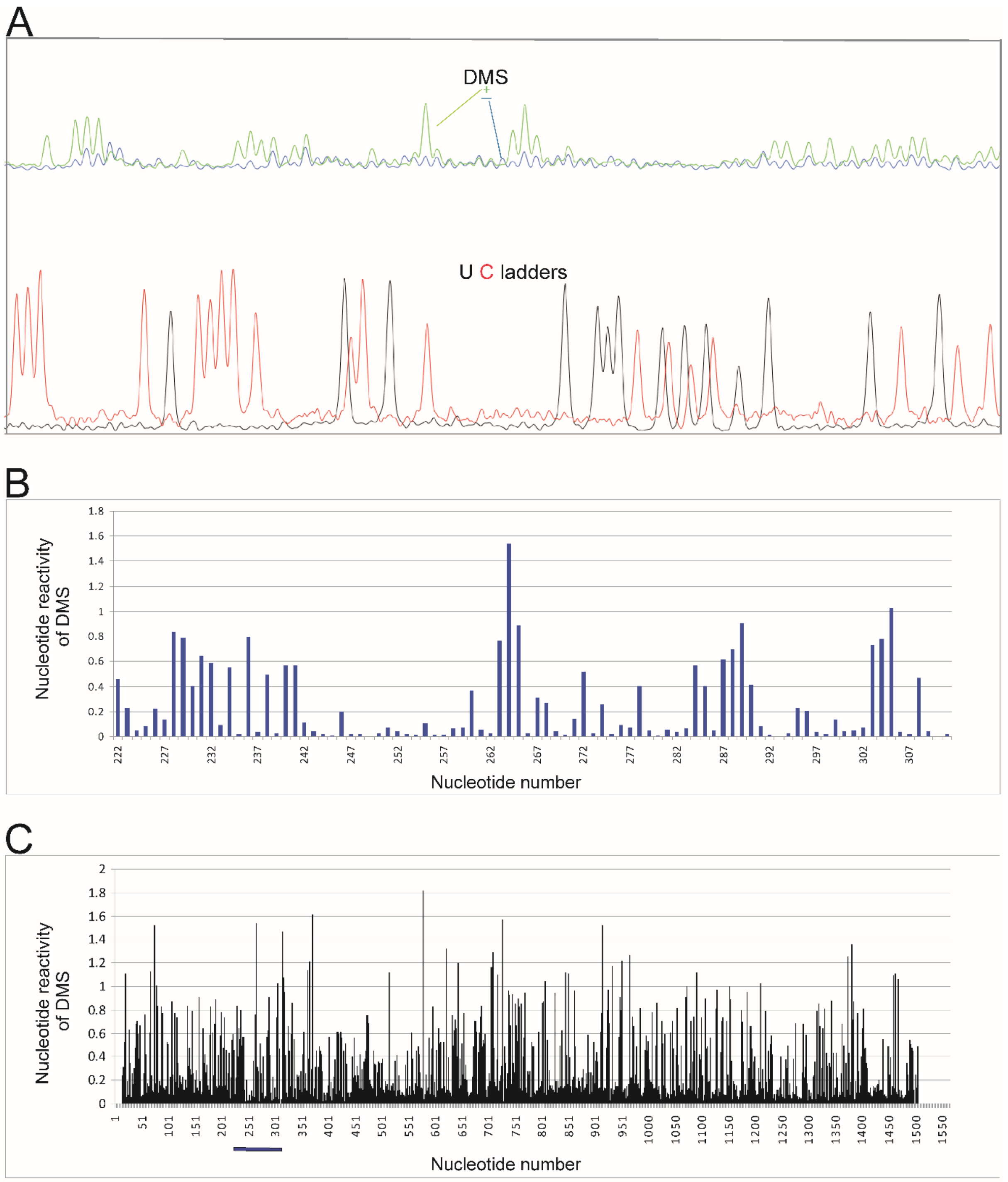

Figure S2. Chemical modifications data at 23°C with DMS for (+)RNA5 detected by reverse transcription with labeled primers followed by capillary electrophoresis. A) - Example of capillary electrophoresis raw data for nt 311-222 (using only Fitted Baseline Adjust option in ShapeFinder program) showing modified RNA (dark green line), unmodified control (light green line) and dideoxy ladders (C – red line and U – black line). B) - (+)RNA5 nucleotides reactivities of DMS mapping for the same fragment showed in panel A, in reverse order (222-311 nt) on the graph. C) - (+)RNA5 nucleotides reactivities of DMS mapping across the entire (+)RNA5 on the graph. The nt region (222-311) showed on panel B is marked by line.

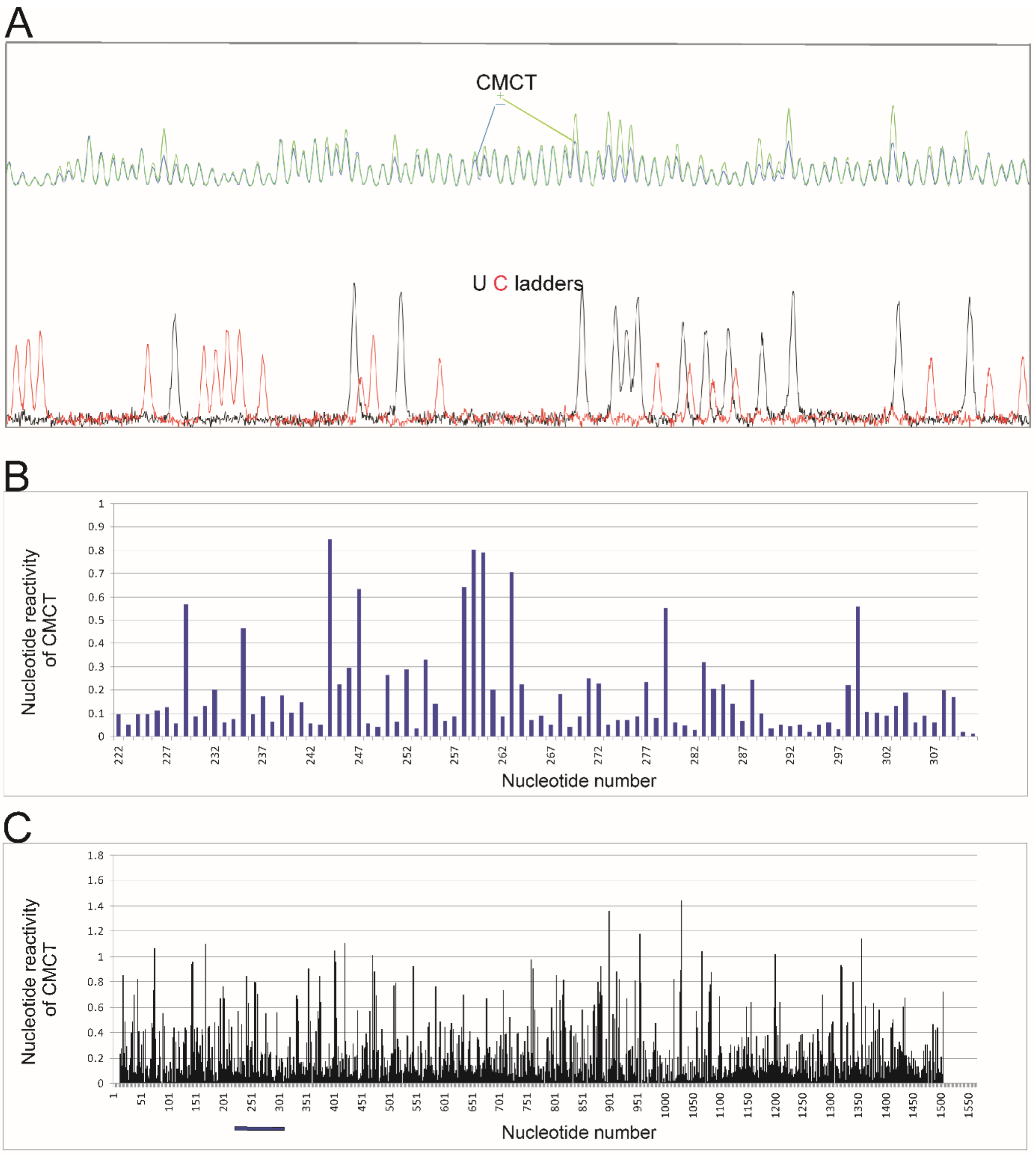

Figure S3. Chemical modifications data at 23°C with CMCT for (+)RNA5 detected by reverse transcription with labeled primers followed by capillary electrophoresis. A) - Example of capillary electrophoresis raw data for nt 311-222 (using only Fitted Baseline Adjust option in ShapeFinder program) showing modified RNA (dark green line), unmodified control (light green line) and dideoxy ladders (C – red line and U – black line). B) - (+)RNA5 nucleotides reactivities of CMCT mapping for the same fragment showed in panel A, in reverse order (222-311 nt) on the graph. C) - (+)RNA5 nucleotides reactivities of CMCT mapping across the entire (+)RNA5 on the graph. The nt region (222-311) showed on panel B is marked by line.

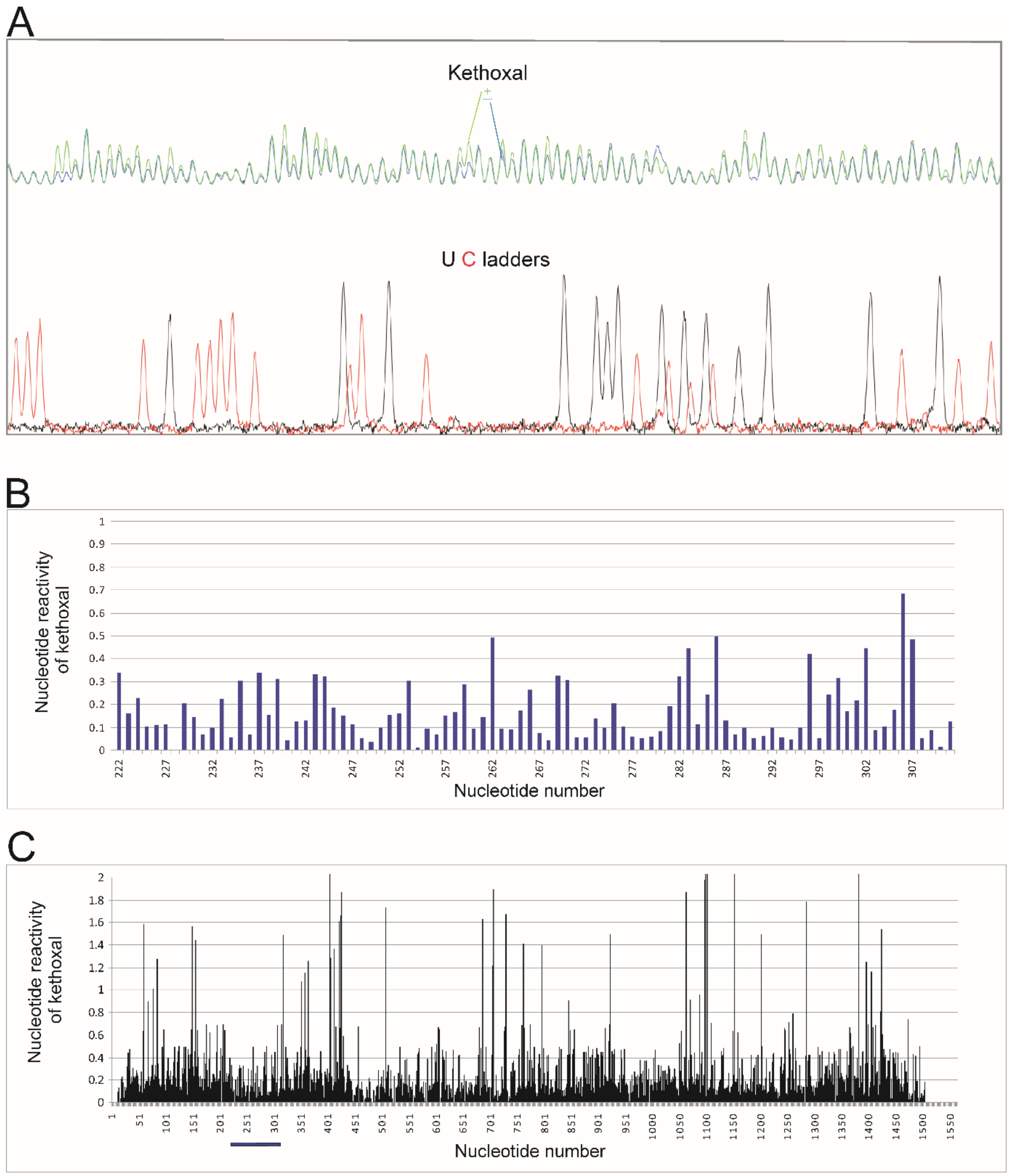

Figure S4. Chemical modifications data at 23°C with kethoxal for (+)RNA5 detected by reverse transcription with labeled primers followed by capillary electrophoresis. A) - Example of capillary electrophoresis raw data for nt 311-222 (using only Fitted Baseline Adjust option in ShapeFinder program) showing modified RNA (dark green line), unmodified control (light green line) and dideoxy ladders (C – red line and U – black line). B) - (+)RNA5 nucleotides reactivities of kethoxal mapping for the same fragment showed in panel A, in reverse order (222-311 nt.) on the graph. C) - (+)RNA5 nucleotides reactivities of kethoxal mapping across the entire (+)RNA5 on the graph. The nt region (222-311) showed on panel B is marked by line.

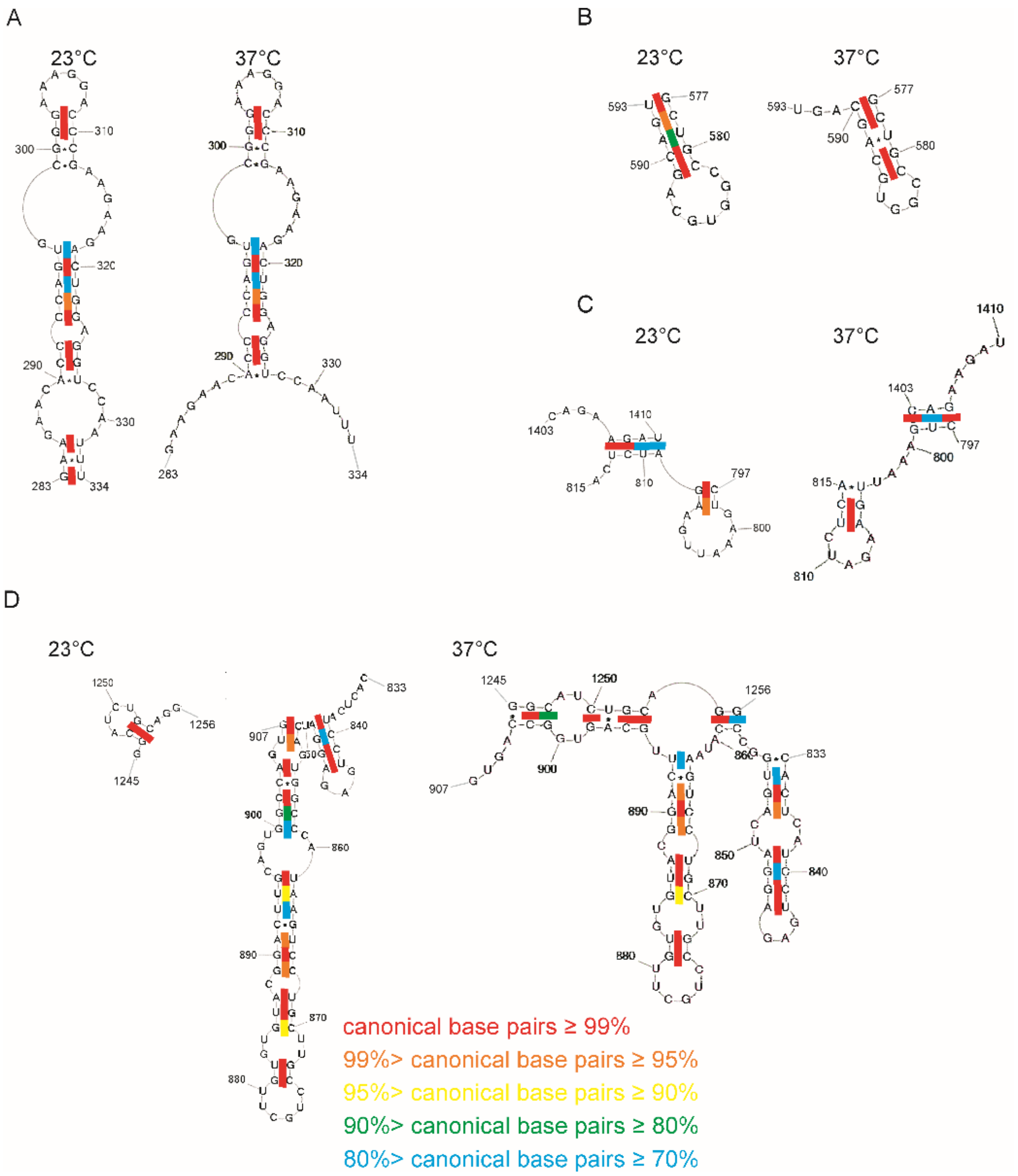

Figure S5. Differences between (+)RNA5 secondary structures predicted based on constraints from 23°C versus 37°C. Differences are in regions: A) - 283-290/332-334; B) - 577-593; C) - 797-815/1403-1410; D) - 833-907/1245-1256.

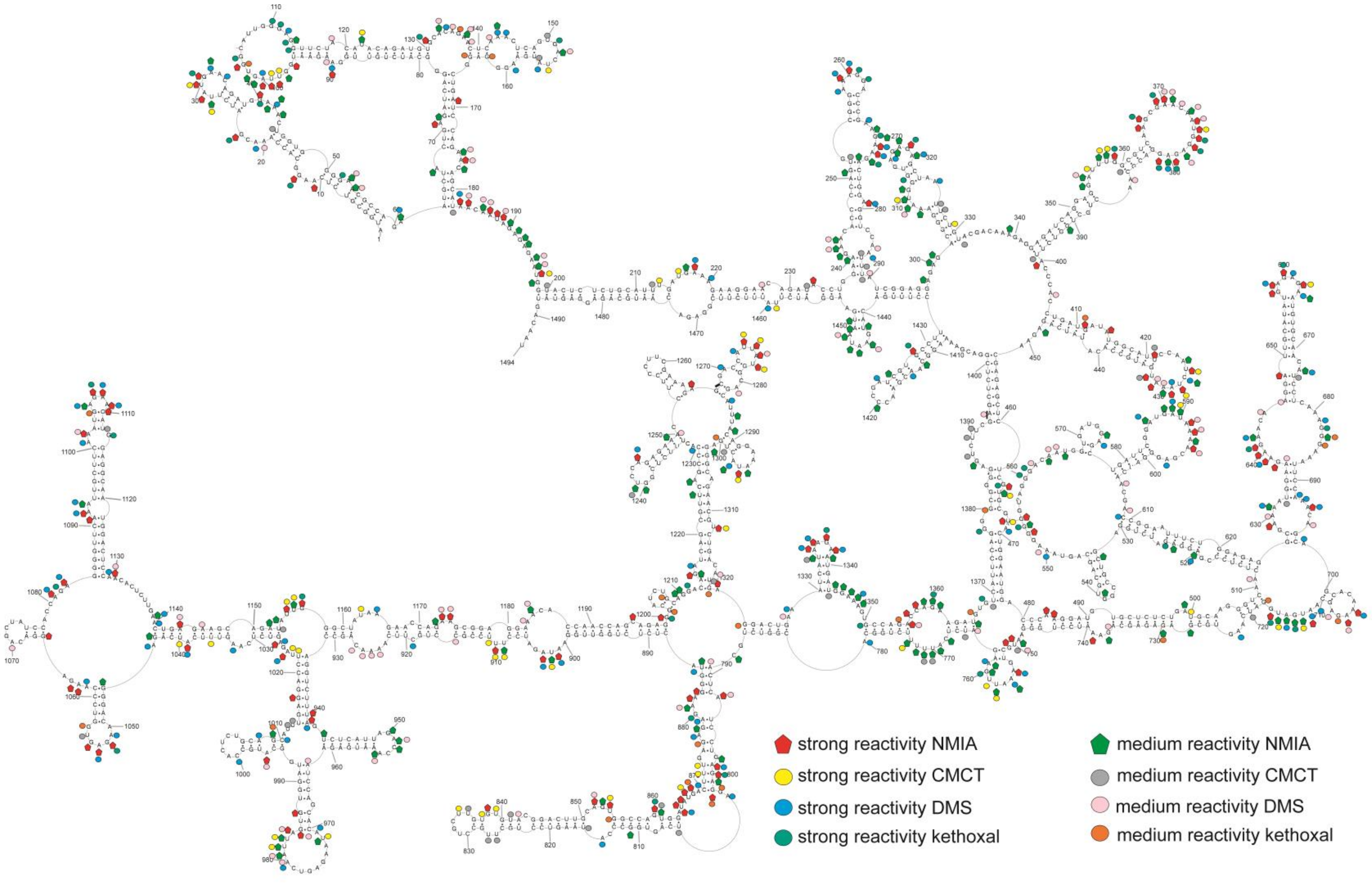

Figure S6. Self-folding of (+)RNA5-ORF predicted by RNAstructure 5.5 using as constraints experimental data obtained at 23°C: strong reactivity of DMS, CMCT, kethoxal and SHAPE reactivities converted to pseudo- free energies. Additionally there are marked medium reactivity of DMS, CMCT and kethoxal. The numbering of (+)RNA5-ORF is from its 5' end. The AUG start codon is nucleotides 1–3..

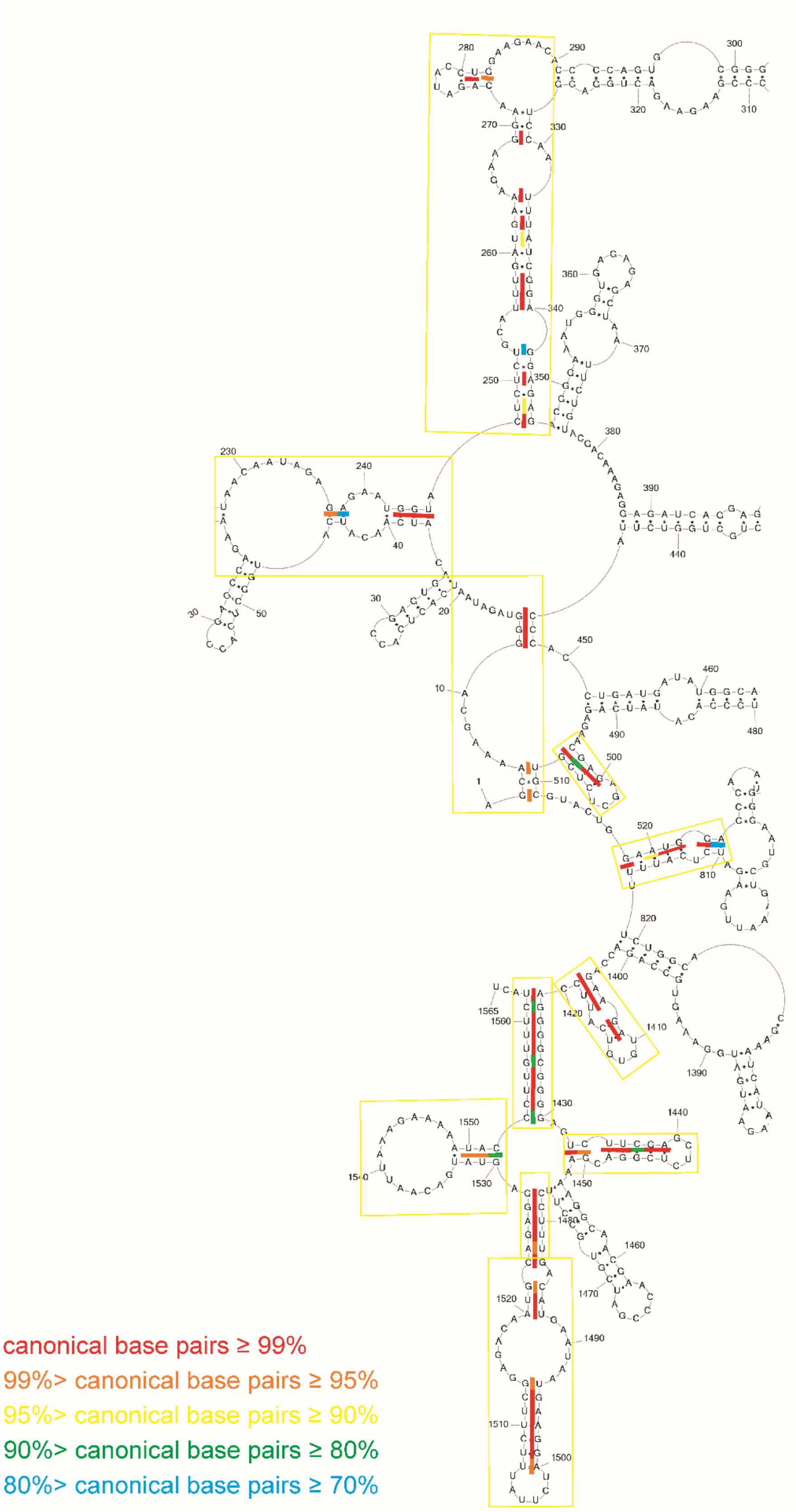

Figure S7. Conservation of (+)RNA5 structure motifs characteristic for constrained secondary structure using a "maximum paring distance 600" parameter in RNAstructure 5.5. Colors indicate percentage of canonical base pairing preserved across type A strains for (+)RNA segment 5. In yellow boxes were indicated different base pairing region comparing to Figure 1.

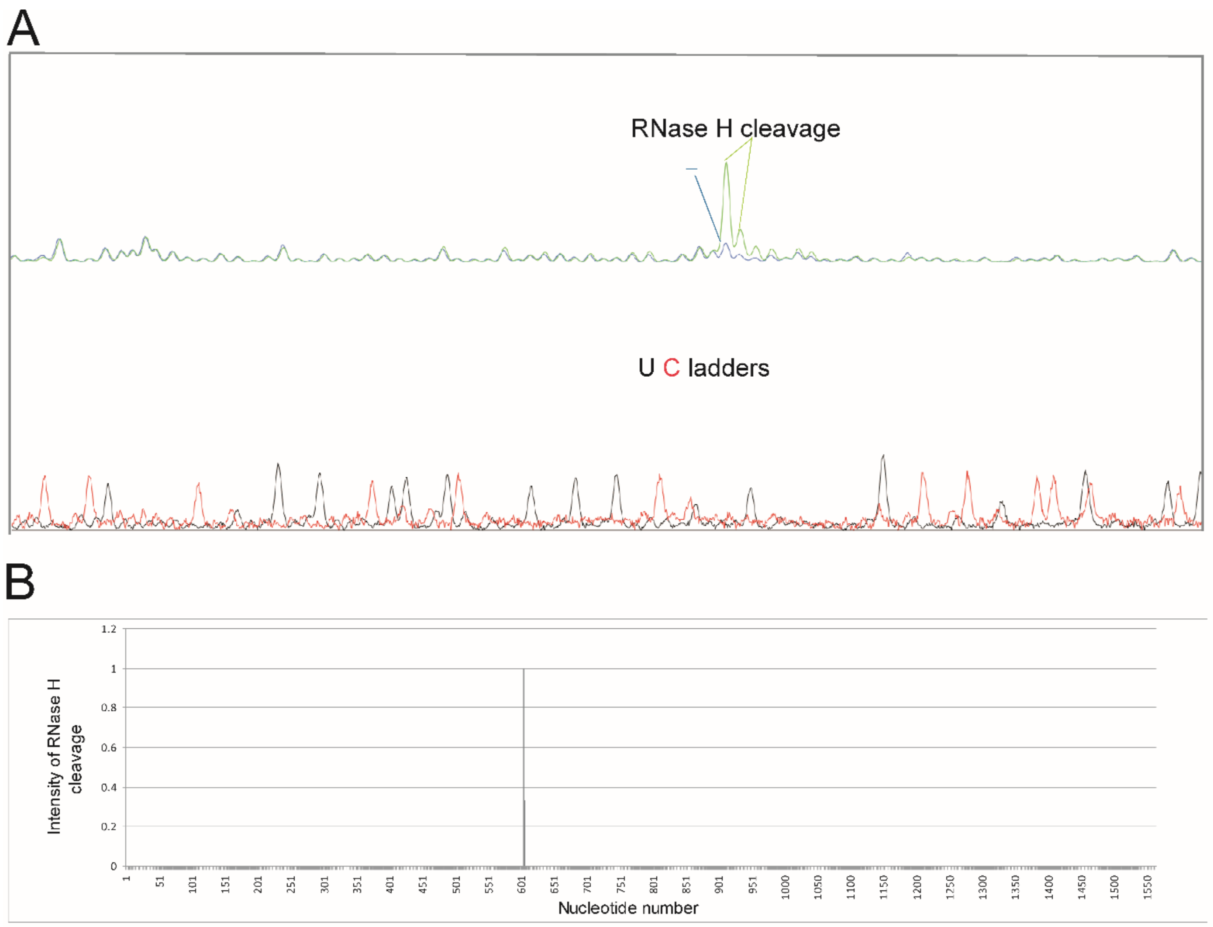

Figure S8. RNase H cleavage of (+)RNA5 with CTACCCC oligonucleotide, detected by reverse transcription with labeled primers followed by capillary electrophoresis. A) - Example of capillary electrophoresis raw data fragment showing region 653-571 nt (using only Fitted Baseline Adjust option in ShapeFinder program) showing RNase H cleavage (green line), control (without DNA oligonucleotide) (blue line) and dideoxy ladders (C – red line and U – black line). B) - Intensity of RNase H cleavage of (+)RNA5 with CTACCCC oligonucleotide on the graph.

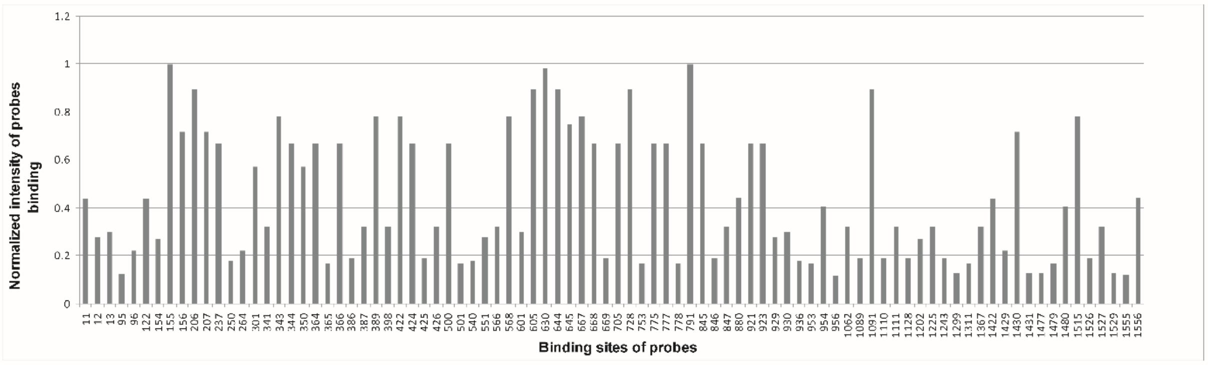

Figure S9. Results of hybridization of (+)RNA5 to isoenergetic microarrays. All complementary sites for probes that bind strongly or moderately are shown. Binding was considered strong (S), medium (M) and weak (W), when the integrated intensities were ≥1/3 (, ≥ 1/9 and ≥ 1/27 of the strongest intensity. In graph bindings were normalized to the strongest intensity and have values in range 1–0, showing bindings: 0.33 ≤ strong and 0.11 ≤ medium <0.33.

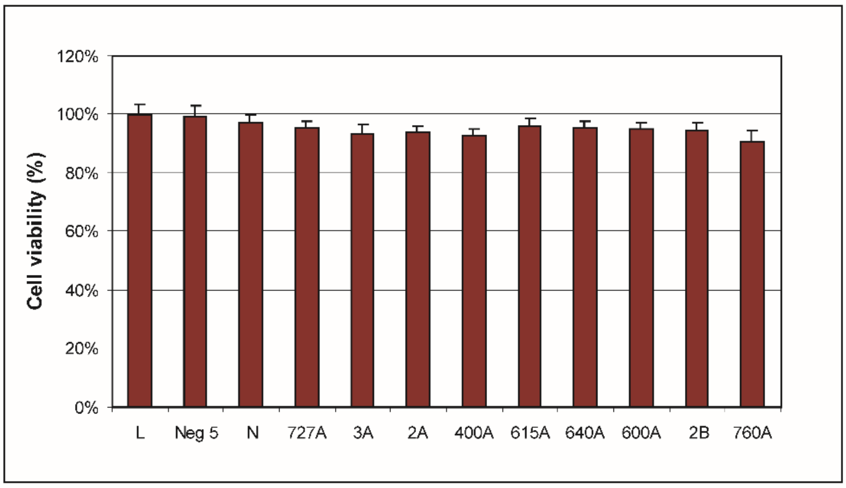

Figure S10. Cell viability measured in MTT test after transfection with 750 nM ASOs. The mean was calculated from three independent experiments, each containing three technical repeats, and the standard deviation is shown. L - MDCK cells treated with Lipofectamine 2000; Neg 5 – MDCK cells transfected with control ASO Neg 5; N - MDCK cells transfected with control ASO N.

References

1. Kierzek, E., Ciesielska, A., Pasternak, K., Mathews, D. H., Turner, D. H., Kierzek, R. The influence of locked nucleic acid residues on the thermodynamic properties of 2'-O-methyl RNA/RNA heteroduplexes. *Nucleic Acids Res.* **33**, 5082-5093 (2005).
2. Pasternak, A., Kierzek, E., Pasternak, K., Fratczak, A., Turner, D. H., Kierzek, R. The thermodynamics of 3'-terminal pyrene and guanosine for the design of isoenergetic 2'-O-methyl-RNA-LNA chimeric oligonucleotide probes of RNA structure. *Biochemistry* **47**, 1249-1258 (2008).
3. Soszynska-Jozwiak, M., Michalak, P., Moss, W. N., Kierzek, R., Kierzek, E. A conserved secondary structural element in the coding region of the influenza A virus nucleoprotein (NP) mRNA is important for the regulation of viral proliferation. *PloS ONE* **10**, e0141132 (2015).
