# Supplementary material for: Influenza virus segment 5 (+)RNA - secondary structure and new targets for antiviral strategies
Source: Sci Rep. 2017 Nov 8;7:15041. doi: 10.1038/s41598-017-15317-5 (PMC5678188; doi:10.1038/s41598-017-15317-5)
Supplement: Supplementary file 4 — Supplementary Data 3 [file 41598_2017_15317_MOESM4_ESM.pdf]

Supplementary Data 3

Influenza virus segment 5 (+)RNA - secondary structure and new targets for antiviral strategies

Marta Soszynska-Jozwiak[1], Paula Michalak[1], Walter N. Moss[2], Ryszard Kierzek[1], Julita Kesy[1], Elzbieta Kierzek[1]*

[1]Institute of Bioorganic Chemistry Polish Academy of Sciences, 61-704 Poznan, Noskowskiego 12/14, Poland

[2]Roy J. Carver Department of Biophysics, Biochemistry and Molecular Biology, Iowa State University, Ames, IA 50011, United States of America

*Corresponding author: E-mail: elzbieta.kierzek@ibch.poznan.pl, Institute of Bioorganic Chemistry Polish Academy of Sciences, 61-704 Poznan, Noskowskiego 12/14, Poland, Tel.: +4861-853-8503; Fax: +4861-852-0532.

Binding site [a]	Probe sequence [b]	ΔG°$_{37}$ of duplex for complementary binding site (kcal/mol)[c]
3	uUgCug	-7.92
4	uUuGcg	-7.76
5	uUuUgg	-5.72
8	uGcUug	-7.92
9	cUgCug	-9.41
10	CcUgC	-9.74
11	CCcUg	-9.22
12	AcCcUg	-12.12
13	UdCcCg	-9.85
14	CUdCcg	-9.12
15	uCuDcg	-8.39
22	GuGdUg	-8.80
23	dGuGdg	-10.06
24	GdGUgg	-9.59
25	UGdGug	-11.04
26	GuGdGg	-9.64
27	GgUgDg	-12.76
28	CgGuGg	-10.37
29	UcGgUg	-12.01
30	CuCgGg	-10.29
31	DcUcGg	-12.16
32	CdCuCg	-10.76
33	uCdCug	-7.53
34	GUcDcg	-8.89
35	uGuCdg	-8.95
36	dUgUcg	-9.25
37	GdUgUg	-9.10
38	uGdUgg	-9.04
41	uGuUgg	-7.96
43	GdUgUg	-9.10
46	CdUgDg	-9.18
47	CCdUgg	-9.22
49	CGcCa	-9.24
50	AcGcC	-9.31
51	GDcGc	-9.09
52	dGdCg	-8.69
53	GdGdCg	-11.92
54	uGdGdg	-10.09
55	uUgDgg	-8.85
56	cUuGdg	-10.82
57	cCuUgg	-9.08
58	GCcUug	-11.61

Binding site [a]	Probe sequence [b]	ΔG°$_{37}$ of duplex for complementary binding site (kcal/mol) [c]
788	CdGgAg	-10.05
789	CCdGg	-9.20
790	CCcDG	-8.17
791	uCcCa	-8.86
792	UUcCcg	-9.17
793	dUuCcg	-8.58
795	GCdUug	-7.03
796	DgCdUg	-9.82
797	CdGcAg	-9.97
798	UCdGcg	-9.63
799	uUcDgg	-8.36
804	uCdDug	-6.75
806	cUuCdg	-8.20
807	uCuUcg	-7.82
809	GdUcUg	-9.02
810	dGdUcg	-9.60
811	GdGdUg	-9.45
812	uGdGdg	-10.09
813	dUgDgg	-9.90
815	dDdUgg	-7.95
818	GdDdDg	-7.79
819	dGdDdg	-9.00
820	CdGdDg	-9.07
821	CCdGdg	-10.27
822	GCcDG	-8.34
823	uGcCa	-9.09
824	GUgCc	-9.41
825	CgUgCg	-10.80
826	CcGuGg	-10.33
827	DcCgUg	-12.20
828	GDcCg	-8.92
829	AgDcC	-8.40
830	CdGdCg	-9.41
831	GCdGdg	-10.44
832	UgCdGg	-10.04
833	GUgCDg	-10.88
834	DgUgCg	-10.74
835	GdGUgg	-9.59
836	UGdGug	-11.04
837	dUgDgg	-9.90
838	GdUGdg	-11.39
839	GGdUgg	-9.69

59	UGcCug	-9.26
60	GUgCc	-9.41
61	gGuGc	-9.68
62	UgGuGg	-9.69
63	uUgGug	-10.89
68	dUcGug	-8.30
69	GdUcGg	-9.48
70	dGdUcg	-12.03
71	DdGdUg	-8.53
72	uDdGdg	-8.78
73	dUdDgg	-8.35
74	CdUdDg	-9.65
75	uCdUdg	-8.16
77	gUuCdg	-8.22
78	uGuUcg	-7.90
79	cUgUug	-7.39
80	UcUgUg	-8.97
81	dUcUgg	-9.23
82	CdUcUg	-10.53
83	CCdUcg	-9.16
84	UcCdUg	-8.97
85	uUcCdg	-8.88
86	uUuCcg	-7.53
90	CCdGug	-8.67
91	DcCdGg	-10.04
92	CdCcAg	-11.10
93	CCdCc	-8.71
94	CCcDC	-7.72
95	CcCcA	-9.88
96	UCcCc	-9.54
97	UUcCcg	-9.17
98	gUuCcg	-9.04
99	cGuUcg	-8.58
100	GcGuUg	-9.23
101	GGcGUg	-9.86
102	uGgCg	-9.88
103	CUgGc	-9.43
104	UcUgGg	-9.91
105	uUcUgg	-11.11
108	GCdUug	-7.03
109	DgCdUg	-9.82
110	UdGCdg	-9.30
111	gUdGcg	-10.29
112	DgUdGg	-9.42
113	CdGuDg	-10.84
114	uCdGug	-8.00

840	DgGdUg	-12.06
841	CdGgAg	-10.05
842	UCdGgg	-9.50
843	CUcDgg	-12.25
844	uCuCdg	-11.00
845	CUcUcg	-9.08
846	CcUcUg	-10.09
847	uCcUcg	-9.99
848	dUcCug	-8.74
849	GDUcCg	-8.60
850	uGdUcg	-8.98
851	CuGdUg	-8.78
852	DcUgDg	-9.71
853	CdCuGg	-8.74
854	CCdCug	-11.28
855	GCcDC	-7.89
856	GGcCA	-8.51
857	GGGCc	-9.41
858	UGgGc	-9.85
859	AuGgGg	-9.25
860	UdUgGg	-11.48
864	GdCuUg	-7.90
865	GgAcUg	-9.76
866	DgGdC	-9.01
867	CdGgAg	-10.05
868	GCdGg	-9.37
869	AgCdGg	-12.43
870	dDgCdg	-12.42
871	CDdGcg	-9.52
872	GcDdGg	-9.87
873	GgCdDg	-12.45
874	AgGcAg	-10.26
875	CdGgC	-9.72
876	GCdGg	-9.37
877	AgCdGg	-12.43
878	dDgCdg	-12.42
879	CDdGcg	-9.52
880	DcDdGg	-8.73
881	CdCdDg	-10.21
882	dCdCDg	-7.72
883	CdCdCg	-8.53
884	dCdCDg	-7.72
885	UdCdCg	-7.82
886	gUdCdg	-8.85
887	CgUdCg	-9.03
888	CcGuDg	-9.94

115	CUcDgg	-9.32
116	uCuCdg	-11.00
117	dUcUcg	-9.17
118	GdUcUg	-9.02
119	uGdUcg	-8.98
120	CuGdUg	-8.78
121	CcUgDg	-10.68
122	CCcUg	-9.22
123	GcCcU	-10.34
124	UGcCc	-9.77
125	AuGcCg	-9.34
126	GdUGcg	-9.82
127	dGdUgg	-9.66
128	CdGdUg	-10.96
129	dCdGdg	-9.83
130	dDcDgg	-9.15
131	cDdCdg	-10.74
132	CcDdCg	-9.25
133	UcCdDg	-9.02
134	uUcCdg	-8.88
135	cUuCcg	-9.02
136	uCuUcg	-7.82
140	cCdUug	-7.39
141	DcCdUg	-9.20
142	dDcCdg	-9.67
143	uDdCcg	-8.72
144	CuDdCg	-7.86
145	DcUdDg	-8.16
146	cDcUdg	-9.07
147	CCdCug	-8.20
148	GCcDC	-7.89
149	uGcCa	-9.09
150	AuGcCg	-9.34
151	DdUgCg	-9.25
152	cDdUgg	-8.44
153	CcDdUg	-10.80
154	CcCdA	-8.23
155	uCcCa	-8.86
156	CUcCc	-9.16
157	CcUcC	-9.51
158	dCcUcg	-10.22
159	dDcCug	-8.48
160	GdAcCg	-9.21
161	DgDdCg	-9.16
162	UdGdDg	-8.36
163	gUdGdg	-9.73

889	uCcGug	-9.12
890	gUcCg	-9.25
891	AgUcCg	-11.78
892	dDgUcg	-9.38
893	CDdGug	-7.36
894	GcDdGg	-9.87
895	uGcDdg	-11.80
896	CuGcDg	-10.12
897	DcUgCg	-10.27
898	CdCuGg	-8.74
899	CCdCug	-11.28
900	GCcDC	-7.89
901	GGcCA	-8.51
902	UGgCc	-9.81
903	CUgGc	-9.43
904	DcUgGg	-10.14
905	CdCuGg	-11.32
906	CCdCug	-11.28
907	UcCdCg	-9.36
908	dUcCdg	-9.93
909	UdUcCg	-8.80
910	dUdUcg	-8.16
912	uCdUdg	-8.16
913	gUcDug	-7.94
914	DgUcDg	-9.69
915	dDgUcg	-9.38
916	dDdGug	-7.40
917	CdDdGg	-8.15
918	uCdDdg	-10.02
919	cUcDdg	-9.06
920	uCuCdg	-8.87
921	CUcUcg	-9.08
922	uCuCug	-7.68
923	CUcUcg	-9.08
924	uCuCug	-7.68
925	uUcUcg	-8.12
926	cUuCug	-7.01
927	cCuUcg	-9.02
928	CcCuUg	-9.50
929	AcCcUg	-10.18
930	UdCcCg	-9.85
931	GUdCcg	-9.14
932	DgUdCg	-8.97
933	GdGUdg	-9.20
934	DgDgUg	-10.26
935	GdGdGg	-10.29

164	UgUdGg	-8.80
167	gUdUgg	-8.68
169	cUgUdg	-9.36
170	UcUgUg	-8.97
171	dUcUgg	-9.23
172	CdUcUg	-10.53
173	dCdUcg	-8.72
174	CdCdUg	-8.14
175	GCdCDg	-8.33
176	UgCdCg	-9.59
177	GUgCDg	-8.86
178	uGuGcg	-10.00
179	cUgUgg	-9.75
180	UcUgUg	-10.91
181	uUcUgg	-8.18
183	dGuUcg	-8.52
185	UGdGug	-7.96
186	uUgDgg	-8.85
191	UGdGug	-7.96
192	CuGdGg	-9.62
193	DcUgDg	-11.73
194	CdCuGg	-8.74
195	uCdCug	-10.61
196	GUcDcg	-8.89
197	DgUcDg	-9.69
198	UdGuCg	-8.44
200	CdUdGg	-8.42
201	uCdUdg	-10.29
203	cUuCdg	-8.20
204	cCuUcg	-9.02
205	CcCuUg	-9.50
206	UcCcUg	-10.47
207	CUcCc	-9.16
208	CcUcC	-9.51
209	GCcUc	-9.33
210	AgCcUg	-10.51
211	CdGcC	-9.68
212	UCdGcg	-9.63
213	dUcDgg	-9.41
214	GdUCdg	-10.90
215	GGdUcg	-9.63
216	UgGdUg	-9.50
217	CUgGdg	-10.37
218	UcUgGg	-9.91
219	uUcUgg	-11.11
221	uGuUcg	-7.90

936	dGdGdg	-12.84
937	CdGdGg	-9.86
938	CCdGdg	-12.40
939	DcCdGg	-10.04
940	dDcCdg	-11.80
941	CdAcCg	-8.78
942	CcDdCg	-9.25
943	UcCdDg	-9.02
944	uUcCdg	-8.88
945	dUuCcg	-8.58
950	GdUcUg	-9.02
951	GGdUcg	-9.63
952	DgGdUg	-10.12
953	dDgGdg	-10.59
954	dDdGgg	-9.43
955	GdDdGg	-11.16
956	gGdDdg	-11.69
957	CgGdAg	-9.74
958	GcGgA	-9.84
959	GGcGG	-9.30
960	AgGcG	-9.51
961	CdGgC	-9.72
962	GCdGg	-9.37
963	AgCdGg	-12.43
964	dDgCdg	-12.42
965	GdAgCg	-9.54
966	uGdDgg	-9.17
969	uUuUgg	-5.72
972	cUgUug	-7.39
973	GcUgUg	-10.34
974	GGcUg	-9.43
975	UgGcUg	-12.68
976	CUgGc	-9.43
977	CcUgG	-9.61
978	dCcUgg	-13.21
979	GDcCUg	-10.67
980	AgDcC	-8.40
981	dDgDcg	-9.58
982	dDdGag	-8.39
985	DcUdDg	-8.16
986	GdCUdg	-8.73
987	dGdCug	-8.64
988	GdGdCg	-9.84
989	uGdGdg	-10.09
990	dUgDgg	-9.90
992	uDdUgg	-7.73

222	cUgUug	-7.39
223	GcUgUg	-10.34
224	uGcUgg	-10.28
225	dUgCug	-12.05
226	UdUgCg	-9.03
235	cUcUdg	-9.28
236	uCuCug	-7.68
237	CUcUcg	-9.08
238	uCuCug	-7.68
239	uUcUcg	-8.12
242	cCdUug	-7.39
243	DcCdUg	-9.20
244	uAcCdg	-8.78
245	GUdCcg	-9.14
246	DgUdCg	-8.97
247	GdGUdg	-9.20
248	DgDgUg	-10.26
249	GdGdGg	-10.29
250	dGdGdg	-12.84
251	CdGdGg	-9.86
252	GCdGdg	-12.57
253	UgCdGg	-10.04
254	dUgCdg	-12.29
255	DdUgCg	-9.25
256	dDdUgg	-7.95
257	CdDdUg	-9.25
258	uCdDdg	-7.89
259	dUcDdg	-8.62
260	CdUcDg	-8.69
261	uCdUcg	-8.49
267	cUuCug	-7.01
268	cCuUcg	-9.02
269	uCcUug	-7.69
270	uUcCug	-7.69
271	gUuCcg	-9.04
272	uGuUcg	-7.90
273	cUgUug	-7.39
274	UcUgUg	-8.97
275	dUcUgg	-9.23
277	gUdUcg	-8.62
278	GgUdUg	-9.14
279	AgGuAg	-8.42
280	CdGgUg	-10.15
281	CCdGg	-9.20
282	UcCdGg	-12.39
283	uUcCdg	-11.01
994	uCuDdg	-8.05
995	gUcUdG	-10.20
996	GgUcUg	-10.15
997	UgGuCg	-9.63
998	uUgGug	-7.81
1003	CUcDug	-7.39
1004	uCuCdg	-8.87
1005	uUcUcg	-8.12
1007	GdUUcg	-7.72
1008	gGdUug	-7.86
1009	UgGdUg	-9.50
1010	CUgGdg	-10.37
1011	GcUgG	-9.78
1012	uGcUgg	-13.21
1013	gUgCug	-12.51
1014	uGuGcg	-10.00
1015	dUgUgg	-9.31
1021	DcUcUg	-9.12
1022	GdCuCg	-9.11
1023	uGdCug	-8.02
1024	uUgDcg	-8.40
1029	DcUdDg	-8.16
1030	cDcUdg	-9.07
1031	dCdCug	-7.76
1032	CdCdCg	-8.53
1033	CCdCDg	-8.16
1034	UcCdCg	-9.36
1035	dUcCdg	-9.93
1036	CdUcCg	-9.51
1037	CCdUcg	-9.16
1038	GcCDUg	-9.00
1039	uGcCa	-9.09
1040	AuGcCg	-9.34
1041	CdUgCg	-9.74
1042	GCdUgg	-9.39
1043	GgCdUg	-12.32
1044	uGgCa	-9.13
1045	GUgGc	-9.45
1046	DgUgGg	-10.61
1047	GdGUgg	-12.52
1048	DgDgUg	-12.20
1049	CdGdGg	-9.86
1050	GCdGdg	-12.57
1051	UgCdGg	-10.04
1052	CuGcDg	-12.14
1053	GcUgCg	-11.41

284	cUuCcg	-9.02
285	uCuUcg	-7.82
288	uGuUcg	-7.90
290	GgUgUg	-10.23
291	GgGUGg	-9.59
292	GgGgU	-10.31
293	UGgGg	-9.72
294	CUgGg	-9.30
295	DcUgGg	-12.72
296	CdCuGg	-11.32
297	GCdCug	-11.45
298	CgCdCg	-10.27
299	CcGcA	-9.59
300	CCcGC	-9.22
301	uCcCg	-9.61
302	UUcCcg	-11.60
303	uUuCcg	-7.53
306	uCcUug	-7.69
307	GUcCug	-8.67
308	gGuCc	-9.45
309	GgGuC	-9.31
310	CgGgU	-9.81
311	uCgGg	-9.69
312	uUcGgg	-12.21
313	cUuCgg	-11.49
314	uCuUcg	-10.25
316	cUuCug	-7.01
317	uCuUcg	-7.82
319	DgUcUg	-9.59
320	CdGuCg	-9.15
321	CCdGug	-8.67
322	UcCdGg	-9.81
323	CUcCdg	-11.97
324	CcUcC	-9.51
325	dCcUcg	-10.22
326	GDcCUg	-8.73
327	GGdCc	-9.18
328	UgGdCg	-9.89
329	uUgGdg	-9.41
330	dUuGgg	-8.68
335	GdUdDg	-8.06
336	cGdUdg	-9.33
337	CcGdUg	-10.14
338	UcCgDg	-10.54
339	CuCcGg	-10.25
340	CcUcC	-9.51

1054	uGcUgg	-10.28
1055	dUgCug	-12.05
1056	DdUgCg	-9.25
1057	dDdUgg	-7.95
1058	CdDdUg	-9.25
1059	uCdDdg	-7.89
1060	cUcDdg	-9.06
1061	CcUcDg	-10.19
1062	uCcUcg	-9.99
1063	GUcCug	-8.67
1064	gGuCc	-9.45
1065	AgGuCg	-9.44
1066	dDgGug	-8.99
1067	uDdGgg	-9.21
1068	CuDdGg	-10.89
1069	uCuDdg	-10.18
1070	cUcUdg	-9.28
1071	DcUcUg	-9.12
1072	GdCuCg	-9.11
1073	dGdCug	-8.64
1074	GdGdCg	-9.84
1075	uGdGdg	-10.09
1076	uUgDgg	-8.85
1077	cUuGdg	-10.82
1079	dDcUug	-9.69
1080	dDdCug	-6.93
1081	GdDdCg	-8.13
1082	uGdDdg	-8.38
1083	dUgDdg	-9.11
1084	GdUGdg	-9.26
1085	uGdUgg	-9.04
1086	CuGdUg	-10.72
1087	uCuGdg	-9.36
1088	CUcUgg	-9.14
1089	CcUcUg	-12.03
1090	CcCuC	-9.21
1091	UcCcUg	-10.47
1092	GUcCc	-9.18
1093	uGuCcg	-9.77
1094	uUgUcg	-8.20
1098	DcUcUg	-9.12
1099	CdCuCg	-8.68
1100	CCdCug	-8.20
1101	DcCdCg	-9.59
1102	GDcCAg	-8.44
1103	GGdCc	-9.18

341	uCcUcg	-9.99
342	CuCcUg	-9.79
343	UcUcCg	-9.81
344	CUcUcg	-9.08
345	uCuCug	-7.68
346	gUcUcG	-10.83
347	CgUcUg	-9.65
348	CcGuCg	-10.27
349	cCcGU	-8.68
350	uCcCg	-9.61
351	UUcCcg	-11.60
352	uUuCcg	-7.53
355	cCdUug	-7.39
356	CcCdUg	-10.17
357	AcCcAg	-9.89
358	CdCcC	-9.06
359	UcDcC	-8.10
360	CUcDcg	-8.87
361	uCuCdg	-8.87
362	CUcUcg	-9.08
363	uCuCug	-7.68
364	CUcUcg	-9.08
365	GcUcUg	-10.26
366	DGcUcg	-10.31
367	UdGcUg	-9.55
368	uUdGcg	-8.78
372	DgDdUg	-8.77
373	CdGdDg	-9.07
374	dCdGdg	-9.83
375	UdCdGg	-8.27
376	gUdCdg	-10.98
377	CgUdCg	-9.03
378	uCgUdg	-9.22
379	gUcGuG	-10.61
380	uGuCgg	-9.31
381	uUgUcg	-10.63
386	CcUcUg	-10.09
387	uCcUcg	-9.99
388	CuCcUg	-9.79
389	UcUcCg	-9.81
390	dUcUcg	-9.17
391	GdUcUg	-9.02
392	uGdUcg	-8.98
393	CuGdUg	-8.78
394	CcUgDg	-10.68
395	uCcUgg	-10.05

1104	GGgDc	-9.46
1105	UgGgAg	-10.53
1106	UUgGgg	-9.31
1107	cUuGgg	-12.05
1110	CcUcUg	-10.09
1111	uCcUcg	-9.99
1112	GUcCug	-8.67
1113	uGuCcg	-9.77
1114	cUgUcg	-9.69
1115	GcUgUg	-10.34
1116	DGcUgg	-10.37
1117	UdGcUg	-11.49
1118	dUdGcg	-9.83
1119	GdUdGg	-8.85
1120	gGdUdg	-11.96
1121	UgGdUg	-9.50
1122	GUgGdg	-10.39
1123	gGuGg	-9.55
1124	UgGuGg	-12.27
1125	cUgGug	-12.38
1126	UcUgGg	-9.91
1127	CUcUgg	-12.07
1128	CcUcUg	-12.03
1129	CcCuC	-9.21
1130	CcCcU	-10.17
1131	AcCcC	-9.60
1132	dDcCc	-8.99
1133	GdAcCg	-9.21
1134	uGdDcg	-8.72
1140	GcDdUg	-9.03
1141	DgCdDg	-9.87
1142	dDgCdg	-10.29
1143	GdAgCg	-9.54
1144	uGdDgg	-9.17
1150	CUcDug	-7.39
1151	uCuCdg	-8.87
1152	uUcUcg	-8.12
1154	uGuUcg	-7.90
1156	CdUgUg	-8.67
1157	CCdUgg	-9.22
1158	UcCdUg	-10.91
1159	CUcCdg	-9.84
1160	CcUcC	-9.51
1161	GCcUc	-9.33
1162	UGcCug	-9.26
1163	uUgCcg	-9.93

396	CuCcUg	-11.73
397	CcUcC	-9.51
398	uCcUcg	-9.99
399	dUcCug	-8.74
400	DdUcCg	-9.02
401	dDdUcg	-7.89
402	CdDdUg	-7.31
403	CcDdDg	-8.91
404	GcCdAg	-9.90
405	CGcCa	-9.24
406	AcGcC	-9.31
407	GDcGc	-9.09
408	UgDcGg	-9.63
409	uUgDcg	-10.83
410	cUuGdg	-8.69
411	gCuUgg	-9.25
412	cGcUug	-11.68
413	UcGcUg	-10.18
414	uUcGcg	-9.41
415	gUuCgg	-8.58
416	uGuUcg	-10.33
420	cCdUug	-10.47
421	UcCdUg	-8.97
422	CUcCdg	-9.84
423	UcUcCg	-9.81
424	CUcUcg	-9.08
425	CcUcUg	-10.09
426	uCcUcg	-9.99
427	GUcCug	-8.67
428	CgUcCg	-10.57
429	GcGuC	-8.94
430	uGcGug	-9.35
431	uUgCgg	-9.47
432	gUuGcg	-11.70
433	dGuUgg	-8.58
435	GCdGug	-8.84
436	AgCdGg	-9.85
437	CdGcAg	-11.72
438	CCdGc	-9.33
439	DcCdGg	-10.04
440	GDcCAg	-10.19
441	AgDcC	-8.40
442	dDgDcg	-9.58
443	uDdGdg	-8.78
444	GuDdGg	-8.33
445	GgUdDg	-11.21

1164	dUuGcg	-8.81
1166	cCdUug	-10.47
1167	UcCdUg	-8.97
1168	GUcCDg	-8.63
1169	AgUcCg	-9.70
1170	GdGUcg	-9.53
1171	GgDgUg	-10.82
1172	UgGdGg	-10.34
1173	uUgGdg	-11.54
1174	gUuGgg	-9.14
1175	uGuUgg	-10.89
1177	DgUgUg	-9.67
1178	GdGUgg	-9.59
1179	DgDgUg	-12.20
1180	dDgDgg	-10.03
1181	cDdGdg	-11.62
1182	uCdDgg	-8.68
1184	gUuCdg	-8.22
1185	dGuUcg	-8.52
1187	uCdGug	-8.00
1188	CUcDgg	-9.32
1189	uCuCdg	-11.00
1190	uUcUcg	-8.12
1191	cUuCug	-7.01
1192	gCuUcg	-9.19
1193	uGcUug	-7.92
1194	cUgCug	-9.41
1195	UcUgCg	-10.04
1196	dUcUgg	-9.23
1198	dUdUcg	-8.16
1200	cDdUdg	-8.05
1201	CcDdUg	-8.86
1202	CcCdA	-8.23
1203	GCcCA	-8.47
1204	AgCcC	-9.93
1205	UdGcC	-8.97
1206	dUdGcg	-9.83
1213	gGuUcg	-9.08
1214	UgGuUg	-8.42
1215	cUgGug	-9.30
1216	UcUgGg	-9.91
1217	uUcUgg	-11.11
1218	cUuCug	-10.09
1219	gCuUcg	-9.19
1220	cGcUug	-8.60
1221	CcGcU	-9.88

446	GgGuAg	-9.79
447	UgGgUg	-10.63
448	gUgGg	-9.85
449	gGuGg	-9.55
450	AgGuGg	-12.08
451	CdGgUg	-12.09
452	UCdGgg	-9.50
453	dUcDgg	-12.34
454	CdUcDg	-10.71
455	uCdUcg	-8.49
457	UdUcDg	-7.98
458	dUdUcg	-8.16
460	cCdUdg	-9.36
461	GcCDUg	-9.00
462	uGcCa	-9.09
463	AuGcCg	-9.34
464	DdUgCg	-9.25
465	GDdUgg	-8.34
466	GgDdUg	-11.27
467	uGgDdg	-9.97
468	uUgGdg	-9.41
469	dUuGgg	-8.68
470	GdUUgg	-10.71
471	dGdUug	-10.38
479	CdUcDg	-8.69
480	GCdUcg	-9.33
481	GgCdUg	-10.38
482	uGgCa	-9.13
483	GUgGc	-9.45
484	uGuGgg	-9.87
485	dUgUgg	-12.24
487	dUdUgg	-8.22
489	uGdUdg	-8.65
490	CuGdUg	-8.78
491	uCuGdg	-9.36
492	CUcUgg	-9.14
493	uCuCug	-10.76
494	uUcUcg	-8.12
496	cGuUcg	-8.58
498	CuCgUg	-9.35
499	UcUcGg	-9.35
500	CUcUcg	-11.51
501	GcUcUg	-10.26
502	DGcUcg	-10.31
503	GdGcUg	-10.69
504	dGdGCg	-10.04

1222	UcCgC	-9.60
1223	CuCcGg	-10.25
1224	CcUcC	-9.51
1225	uCcUcg	-9.99
1226	uUcCug	-7.69
1227	uUuCcg	-7.53
1231	GgUgUg	-10.23
1232	UgGuGg	-9.69
1233	uUgGug	-10.89
1234	gUuGgg	-9.14
1235	gGuUgg	-12.07
1236	UgGuUg	-10.36
1237	cUgGug	-9.30
1238	GcUgG	-9.78
1239	uGcUgg	-13.21
1240	cUgCug	-12.49
1241	UcUgCg	-10.04
1242	CUcUgg	-9.14
1243	CcUcUg	-12.03
1244	CcCuC	-9.21
1245	GcCcU	-10.34
1246	UGcCc	-9.77
1247	AuGcCg	-9.34
1248	GdUGcg	-9.82
1249	dGdUgg	-9.66
1250	CdGdUg	-10.96
1251	GCdGdg	-10.44
1252	UgCdGg	-10.04
1253	CuGcDg	-12.14
1254	CcUgC	-9.74
1255	uCcUgg	-10.05
1256	GUcCug	-11.75
1257	uGuCcg	-9.77
1258	cUgUcg	-9.69
1259	UcUgUg	-8.97
1260	dUcUgg	-9.23
1261	GdUcUg	-10.96
1262	uGdUcg	-8.98
1263	CuGdUg	-8.78
1264	GcUgDg	-10.85
1265	CgCuGg	-10.48
1266	DcGcUg	-12.35
1267	DdCGcg	-9.43
1268	GdDcGg	-9.22
1269	uGdDcg	-11.15
1271	GcUgDg	-10.85

505	GdGdGg	-10.29
506	CGdGdg	-12.37
507	DcGdGg	-10.01
508	CdCgDg	-11.73
509	GCdCg	-8.42
510	CgCdCg	-12.35
511	dCgCdg	-10.69
512	UdCgCg	-9.56
513	GUdCgg	-8.68
514	DgUdCg	-11.05
515	CdGuDg	-8.82
516	CCdGug	-8.67
517	UcCdGg	-9.81
518	uUcCdg	-11.01
519	dUuCcg	-8.58
521	cCdUug	-7.39
522	UcCdUg	-8.97
523	GUcCDg	-8.63
524	gGuCc	-9.45
525	GgGuC	-9.31
526	UgGgUg	-10.63
527	UUgGgg	-9.31
528	cUuGgg	-12.05
529	cCuUgg	-12.01
530	uCcUug	-10.77
531	dUcCug	-8.74
532	CdUcCg	-9.51
533	dCdUcg	-8.72
534	CdCdUg	-8.14
535	GCdCDg	-8.33
536	AgCdCg	-9.40
537	GdGCdg	-10.44
538	dGdGCg	-10.04
539	GdGdGg	-10.29
540	dGdGdg	-12.84
541	CdGdGg	-9.86
542	uCdGdg	-11.73
543	dUcDgg	-9.41
544	CdUcDg	-10.71
545	GCdUcg	-9.33
546	UgCdUg	-9.20
547	uUgCdg	-9.11
548	cUuGcg	-9.25
549	cCuUgg	-9.08
550	CcCuUg	-11.44
551	AcCcUg	-10.18

1272	GGcUg	-9.43
1273	GgGcU	-10.42
1274	UGgGc	-9.85
1275	gUgGg	-9.85
1276	DgUgGg	-13.19
1277	dDgUgg	-12.37
1278	dDdGug	-10.48
1279	GdDdGg	-8.58
1280	dGdDdg	-11.13
1281	GdGdDg	-9.50
1282	CGdGdg	-10.24
1283	CcGdG	-9.48
1284	ACcGdg	-12.03
1285	GDcCg	-8.92
1286	GGdCc	-9.18
1287	UgGdCg	-9.89
1288	CUgGdg	-10.37
1289	UcUgGg	-9.91
1290	CUcUgg	-12.07
1291	uCuCug	-10.76
1292	uUcUcg	-8.12
1296	dGgUug	-8.43
1297	dDgGug	-8.99
1298	GdAgGg	-9.41
1299	GgDdGg	-12.75
1300	GgGdAg	-11.99
1301	AgGgAg	-10.34
1302	dDgGg	-9.52
1303	GdAgGg	-11.99
1304	CGdDgg	-12.25
1305	UcGdAg	-10.25
1306	uUcGdg	-8.85
1311	GcUcUg	-10.26
1312	CgCuCg	-10.42
1313	UcGcUg	-10.18
1314	gUcGc	-9.42
1315	GgUcGg	-10.61
1316	UgGuCg	-11.71
1317	dUgGug	-8.86
1318	DdUgGg	-9.12
1319	uDdUgg	-10.66
1321	CdUdDg	-7.63
1322	cCdUdg	-9.36
1323	GcCDUg	-9.00
1324	uGcCa	-9.09
1325	CUGcCg	-9.66

552	GAcCc	-8.79
553	UgAcCg	-9.50
554	uUgDcg	-8.40
555	gUuGdg	-8.71
556	dGuUgg	-8.58
558	DgDgUg	-10.26
559	GdGdGg	-10.29
560	GgDgAg	-12.47
561	GGgDg	-9.91
562	CgGgA	-9.71
563	uCgGg	-9.69
564	CuCgGg	-12.87
565	CcUcGg	-13.13
566	uCcUcg	-12.42
567	CuCcUg	-9.79
568	UcUcCg	-9.81
569	dUcUcg	-9.17
570	GdUcUg	-9.02
571	dGdUcg	-9.60
572	CdGdUg	-9.02
573	CCdGdg	-10.27
574	UcCdGg	-9.81
575	CUcCdg	-11.97
576	gCuCc	-9.56
577	DGcUcg	-10.31
578	CdGcUg	-10.26
579	GCdGc	-9.50
580	GGCdGg	-9.64
581	CgGcA	-9.63
582	CCgGC	-9.26
583	ACcGg	-8.83
584	cDcCg	-9.02
585	GCdCc	-8.88
586	UgCdCg	-9.59
587	CuGcDg	-10.12
588	GcUgCg	-11.41
589	uGcUgg	-10.28
590	cUgCug	-12.49
591	DcUgCg	-10.27
592	UdCUgg	-7.98
597	CcCuUg	-9.50
598	CcCcU	-10.17
599	CCCcC	-9.51
600	AcCcC	-9.60
601	UdCcCg	-9.85
602	CUdCcg	-9.12

1326	GcUgCg	-11.41
1327	uGcUgg	-10.28
1328	dUgCug	-12.05
1329	DdUgCg	-9.25
1330	dDdUgg	-7.95
1332	gUdDdg	-8.02
1334	cUgUdg	-9.36
1335	CcUgUg	-10.17
1336	uCcUgg	-10.05
1337	uUcCug	-10.77
1338	uUuCcg	-7.53
1342	DgUdUg	-8.58
1343	CdGuDg	-8.82
1344	uCdGug	-8.00
1345	CUcDgg	-9.32
1346	CcUcDg	-12.21
1347	CcCuC	-9.21
1348	GcCcU	-10.34
1349	UGcCc	-9.77
1350	CUGcCg	-9.66
1351	UcUgCg	-10.04
1352	uUcUgg	-8.18
1354	cGuUcg	-8.58
1355	dCgUug	-7.48
1356	GdCgUg	-9.63
1357	dGdCg	-8.69
1358	CdGdCg	-11.49
1359	uCdGdg	-9.60
1360	GUcDgg	-9.34
1361	uGuCdg	-11.08
1362	dUgUcg	-9.25
1363	CdUgUg	-8.67
1364	uCdUgg	-8.55
1365	CUcDug	-10.47
1366	CcUcDg	-10.19
1367	uCcUcg	-9.99
1368	GUcCug	-8.67
1369	AgUcCg	-9.70
1370	CdGuCg	-9.15
1371	uCdGug	-8.00
1372	uUcDgg	-8.36
1378	UdUgDg	-8.47
1387	CdUcDg	-8.69
1388	CCdUcg	-9.16
1389	UcCdUg	-8.97
1390	uUcCdg	-8.88

603	CcUdCg	-9.47
604	CcCuAg	-9.69
605	UcCcUg	-10.47
606	GUcCc	-9.18
607	uGuCcg	-9.77
608	uUgUcg	-8.20
611	cCdUug	-10.47
612	DcCdUg	-9.20
613	CdCcAg	-9.35
614	UcDcC	-8.10
615	dUcDcg	-8.96
616	CdUcDg	-8.69
617	CCdUcg	-9.16
618	UcCdUg	-8.97
619	CUcCdg	-9.84
620	gCuCc	-9.56
621	DGcUcg	-10.31
622	CdGcUg	-10.26
623	UCdGcg	-9.63
624	dUcDgg	-9.41
625	DdUcDg	-10.22
626	GDdUcg	-8.28
627	cGdDug	-7.92
628	CcGdAg	-9.70
629	UcCgDg	-10.54
630	AuCcGg	-8.65
631	CdUcCg	-11.59
632	uCdUcg	-8.49
634	UdUcDg	-7.98
641	CuCgUg	-9.35
642	CcUcGg	-10.55
643	CcCuC	-9.21
644	UcCcUg	-10.47
645	AuCcCg	-9.11
646	GDUcCg	-8.60
647	uGdUcg	-8.98
649	gUuGdg	-8.71
650	cGuUgg	-8.64
652	gUcGuG	-10.61
653	GgUcGg	-10.61
654	CgGuC	-8.81
655	CcGgU	-9.77
656	UCcGg	-9.12
657	uUcCgg	-12.17
658	dUuCcg	-11.01
662	dGdDdg	-9.00

1391	uUuCcg	-7.53
1395	GCdCug	-8.37
1396	GGCdCg	-9.19
1397	uGgCa	-9.13
1398	CUgGc	-9.43
1399	UcUgGg	-9.91
1400	gUcUgg	-12.62
1401	GgUcUg	-12.09
1402	UgGuCg	-9.63
1403	cUgGug	-9.30
1404	UcUgGg	-9.91
1405	uUcUgg	-11.11
1406	cUuCug	-10.09
1407	uCuUcg	-7.82
1409	CdUcUg	-8.59
1410	dCdUcg	-8.72
1411	CdCdUg	-8.14
1412	dCdCDg	-7.72
1413	GdCdCg	-8.96
1414	uGdCdg	-9.21
1415	dUgDcg	-9.45
1417	GDdUgg	-8.34
1418	GgDdUg	-11.27
1419	uGgDdg	-9.97
1420	CUgGdg	-10.37
1421	CcUgG	-9.61
1422	CCcUg	-9.22
1423	CcCcU	-10.17
1424	GCCCc	-9.33
1425	CGcCC	-9.22
1426	CcGCC	-9.22
1427	CCcGC	-9.22
1428	CCcCG	-9.05
1429	UCcCc	-9.54
1430	CUcCc	-9.16
1431	DcUcCg	-10.04
1432	GdCuCg	-9.11
1433	dGdCug	-8.64
1434	dDgDcg	-9.58
1435	GdDgDg	-9.74
1436	CGdDgg	-9.32
1437	UcGdAg	-10.25
1438	CuCgDg	-9.86
1439	gCuCg	-9.10
1440	DGcUcg	-12.74
1441	GdGcUg	-10.69

663	CdGdDg	-9.07
664	CCdGdg	-10.27
665	UcCdGg	-9.81
666	CUcCdg	-11.97
667	UcUcCg	-9.81
668	CUcUcg	-9.08
669	CcUcUg	-10.09
670	GCcUc	-9.33
671	cGcCU	-8.83
672	UcGcC	-9.60
673	uUcGcg	-9.41
678	cCdUug	-7.39
679	UcCdUg	-8.97
680	uUcCdg	-8.88
681	cUuCcg	-9.02
682	uCuUcg	-7.82
684	cUuCug	-7.01
685	uCuUcg	-7.82
688	uGuUcg	-7.90
691	cCuUgg	-9.08
692	uCcUug	-10.77
693	dUcCug	-8.74
694	DdUcCg	-9.02
695	cDdUcg	-8.38
696	GcDdUg	-9.03
697	uGcDdg	-9.67
698	dUgCdg	-10.16
699	UdUgCg	-9.03
700	dUdUgg	-8.22
702	uCdUdg	-8.16
703	CUcDug	-7.39
704	uCuCdg	-8.87
705	CUcUcg	-9.08
706	uCuCug	-7.68
707	uUcUcg	-8.12
711	CdCdUg	-8.14
712	GCdCDg	-8.33
713	UgCdCg	-9.59
714	uUgCdg	-9.11
715	gUuGcg	-9.27
716	uGuUgg	-7.96
718	GdUgUg	-9.10
719	GGdUgg	-9.69
720	DgGdUg	-12.06
721	GDgGa	-8.87
722	UGdGgg	-9.99

1442	dGdGCg	-10.04
1443	GdGdGg	-10.29
1444	CGdGdg	-12.37
1445	CcGdG	-9.48
1446	UcCgDg	-12.56
1447	gUcCg	-9.25
1448	CgUcCg	-12.65
1449	uCgUcg	-9.55
1450	uUcGug	-7.25
1455	GCcUug	-8.53
1456	UGcCug	-9.26
1457	uUgCcg	-9.93
1458	gUuGcg	-9.27
1459	cGuUgg	-8.64
1461	uUcGug	-7.25
1462	gUuCgg	-8.58
1463	gGuUcg	-11.51
1464	GgGuUg	-9.60
1465	CgGgU	-9.81
1466	uCgGg	-9.69
1467	DuCgGg	-12.43
1468	GdUcGg	-12.06
1469	cGdUcg	-12.09
1470	DcGdUg	-9.17
1471	CdCgDg	-9.71
1472	GCdCg	-8.42
1473	GGCdCg	-11.27
1474	AgGcAg	-10.26
1475	dDgGc	-9.65
1476	GdAgGg	-9.41
1477	GgDdGg	-12.75
1478	DgGdDg	-12.19
1479	dDgGdg	-10.59
1480	dDdGgg	-9.43
1481	CdDdGg	-10.73
1482	uCdDdg	-10.02
1483	gUcDdg	-9.08
1484	uGuCdg	-8.95
1485	dUgUcg	-9.25
1486	CdUgUg	-8.67
1487	uCdUgg	-8.55
1496	cUuCdg	-8.20
1497	cCuUcg	-9.02
1498	uCcUug	-7.69
1499	dUcCug	-8.74
1500	GDUcCg	-8.60

723	uUgDgg	-11.78	1501	dGdUcg	-9.60
727	CcCuUg	-9.50	1502	DdGdUg	-8.53
728	UcCcUg	-10.47	1503	uDdGdg	-8.78
729	UUcCcg	-9.17	1504	dUdDgg	-8.35
730	uUuCcg	-7.53	1508	dGdDdg	-9.00
734	GgDdUg	-9.33	1509	dDgDdg	-9.24
735	uGgDdg	-9.97	1510	GdDgDg	-9.74
736	uUgGdg	-9.41	1511	CGdDgg	-9.32
740	cUgUug	-7.39	1512	CcGdAg	-11.45
741	GcUgUg	-10.34	1513	UcCgDg	-10.54
742	uGcUgg	-10.28	1514	CuCcGg	-10.25
743	cUgCug	-12.49	1515	UcUcCg	-11.89
744	GcUgCg	-11.41	1516	gUcUcG	-10.83
745	uGcUgg	-10.28	1517	uGuCug	-7.76
746	gUgCug	-12.51	1518	uUgUcg	-8.20
747	uGuGcg	-10.00	1521	GCdUug	-10.11
748	uUgUgg	-8.26	1522	UgCdUg	-9.20
753	GcUcUg	-10.26	1523	CuGcDg	-10.12
754	uGcUcg	-10.22	1524	UcUgCg	-10.04
755	uUgCug	-7.92	1525	CUcUgg	-9.14
756	dUuGcg	-8.81	1526	CcUcUg	-12.03
760	CdUcDg	-8.69	1527	uCcUcg	-9.99
761	CCdUcg	-9.16	1528	CuCcUg	-9.79
762	UcCdUg	-8.97	1529	DcUcCg	-10.04
763	dUcCdg	-9.93	1530	UdCUcg	-7.92
764	GDUcCg	-8.60	1532	CdUdCg	-7.97
765	uGdUcg	-8.98	1533	uCdUdg	-8.16
767	cUuGdg	-8.69	1534	gUcDug	-7.94
770	GCdCug	-8.37	1535	uGuCdg	-8.95
771	CgCdCg	-10.27	1536	uUgUcg	-8.20
772	UcGCdg	-9.93	1551	GgUdUg	-9.14
773	CuCgCg	-10.42	1552	GgGuAg	-9.79
774	UcUcGg	-9.35	1553	AgGgUg	-10.44
775	CUcUcg	-11.51	1554	dDgGg	-9.52
776	uCuCug	-7.68	1555	CDdGgg	-12.32
777	CUcUcg	-9.08	1556	DcDdGg	-11.31
778	GcUcUg	-10.26	1557	dDcDdg	-10.49
779	uGcUcg	-10.22	1558	dDdcDg	-8.47
780	cUgCug	-9.41	1559	GdDdCg	-8.13
781	UcUgCg	-10.04	1560	dGdDdg	-9.00
782	uUcUgg	-8.18	1561	UdGdDg	-8.36
786	gGdUug	-7.86	1562	gUdGdg	-9.73
787	DgGdUg	-10.12	1563	DgUdGg	-9.42

List of used probes (pentamers and hexamers) for isoenergetic microarrays with their binding sites in (+)RNA5; a - binding sites of probes, sites are denoted by the middle nucleotide of the complementary RNA region (or two nucleotides for probes with an even number of nucleotides); b - nucleotides in capital letter (A, C, G, U, D) are 2’-O-methyl-RNA nucleotides, in small letter (a, c, g, u, d) are LNA

nucleotides, D and d are 2.6 –diaminopurine (2’-O-methyl type or LNA, respectively); d - $\Delta G°_{37}$ calculated as modified probe/RNA duplex [1,2].

Binding sites [a]	Sequence 5’→3’ [b]	$\Delta G°_{37}$ of duplex for complementary binding site (kcal/mol) [c]
19	UGaUuAuCU	-11.78
229	AUuGuUaUG	-10.99
232	UCuAuUgUU	-12.29
264	UcUuUcA	-10.26
474	CAuUuAgAU	-10.93
476	AUcAuUuAG	-10.73
637	CGuUuUaUC	-11.72
1018	CUcUuAuGU	-13.78
1137	CAaUuUgAA	-10.36
1147	CaUuUgA	-9.16
1209	UUcUuAuAG	-11.32
1375	UgAuUuC	-9.17
1381	AUuCuUaUG	-10.91
1384	UcAuUcU	-10.15
1489	UUaUuCaUG	-10.62
1492	UCaUuAuUC	-10.56
1540	CUuUaAuUG	-10.01
1543	UUuCuUuAA	-9.52
1547	UAuUuUuCU	-9.75

List of used additional, longer probes for isoenergetic microarrays with their binding sites in (+)RNA5**;** a - binding sites of probes. sites are denoted by the middle nucleotide of the complementary RNA region (or two nucleotides for probes with an even number of nucleotides); b – nucleotides in capital letter (A, C, G, U, D) are 2’-O-methyl-RNA nucleotides, in small letter (a, c, g, u, d) are LNA nucleotides; c - $\Delta G°_{37}$ calculated as modified probe/RNA duplex [1,2].

References

1 Kierzek, E., Ciesielska, A., Pasternak, K., Mathews, D. H., Turner, D. H., Kierzek, R. The influence of locked nucleic acid residues on the thermodynamic properties of 2'-O-methyl RNA/RNA heteroduplexes. *Nucleic Acids Res.* **33**, 5082-5093 (2005).

2 Pasternak, A., Kierzek, E., Pasternak, K., Fratczak, A., Turner, D. H., Kierzek, R. The thermodynamics of 3'-terminal pyrene and guanosine for the design of isoenergetic 2'-O-methyl-RNA-LNA chimeric oligonucleotide probes of RNA structure. *Biochemistry* **47**, 1249-1258 (2008).
