# Supplementary material for: Influenza virus segment 5 (+)RNA - secondary structure and new targets for antiviral strategies
Source: Sci Rep. 2017 Nov 8;7:15041. doi: 10.1038/s41598-017-15317-5 (PMC5678188; doi:10.1038/s41598-017-15317-5)
Supplement: Supplementary file 5 — Supplementary File [file 41598_2017_15317_MOESM5_ESM.pdf]

Supplementary Script File

Influenza virus segment 5 (+)RNA - secondary structure and new targets for antiviral strategies

Marta Soszynska-Jozwiak[1], Paula Michalak[1], Walter N. Moss[2], Ryszard Kierzek[1], Julita Kesy[1], Elzbieta Kierzek[1]*

[1]Institute of Bioorganic Chemistry Polish Academy of Sciences, 61-704 Poznan, Noskowskiego 12/14, Poland

[2]Roy J. Carver Department of Biophysics, Biochemistry and Molecular Biology, Iowa State University, Ames, IA 50011, United States of America

*Corresponding author: E-mail: elzbieta.kierzek@ibch.poznan.pl, Institute of Bioorganic Chemistry Polish Academy of Sciences, 61-704 Poznan, Noskowskiego 12/14, Poland, Tel.: +4861-853-8503; Fax: +4861-852-0532.

```
#! Usr/bin/perl -w

# Read FASTA file and bracket structure file for the RNA structure. Output a
# count of the pairs in the alignment. This script is useful for searching for
# compensatory changes that support or contradict a hypothetical RNA structure.
#
# Usage:
#
# perl PairCount.pl "Fasta alignment" "bracket structure file" > "output file"
#
# The outfile has pair i j followed by the number of pairs of
# $GC\$CG\$AU\$UA\$GU\$UG\$GA\$AG\$AA\$GG\$UU\$CC\$AC\$UC\$CA\$CU
# that are found at i-j in the alignment
#
# Author: Walter Moss
# e-mail: wmoss@iastate.edu
# Iowa State University

$infile1 = $ARGV[0];

open (INFILE1, "$infile1") || die "Can't open the infile!\n";

my @BigFasta = <INFILE1>;

close(INFILE1) || die "can't close file";

my $N = @BigFasta;

#Put FASTA seqs into a Hash

my %FASTA = ();
my $CurrentSeq = "";

for ($i = 0; $i < $N; $i++) {

    $Line = $BigFasta[$i];
    chomp $Line;

    #Place title and seq into their respective places in the array FASTA
    if (substr($Line, 0, 1) eq ">") {

        my $SeqName = $Line;
        chomp $SeqName;
        $SeqName =~ s/>//g;
        $SeqName =~ s/:/-/g;
        $CurrentSeq = $SeqName;

        }

    if ( $Line =~ m/(A|G|C|U|T|Y|R|K|M|B|D|H|V|N|S|W|\?|-)/g && substr($Line, 0, 1) ne ">") {
    $Line =~ s/T/U/g;
    $FASTA{$CurrentSeq} .= $Line;

    }
}

$infile2 = $ARGV[1];
$targetfile2 = "$dir/$infile2";
open (INFILE2, "$targetfile2") || die "Can't open the infile!\n";
```

```
my  $Str = <INFILE2>;
close(INFILE2) || die "can't close file";

chomp $Str;
@Str = split("", $Str);

my @Pairs = BasePair (@Str);
my @SeqNames = keys(%FASTA);

# test each position i-j for the types of pair that is found, count pairs

my $Pairlen = @Pairs;

for (my $j=0; $j < $Pairlen; $j += 2) {

   my $LBP = $Pairs[$j];
   my $RBP = $Pairs[$j+1];

   my $AA = 0;
   my $AU = 0;
   my $AG = 0;
   my $AC = 0;
   my $GA = 0;
   my $GU = 0;
   my $GG = 0;
   my $GC = 0;
   my $UA = 0;
   my $UU = 0;
   my $UG = 0;
   my $UC = 0;
   my $CA = 0;
   my $CU = 0;
   my $CG = 0;
   my $CC = 0;

   foreach my $SequenceName (@SeqNames) {

      chomp $SequenceName;
      my $Sequence = $FASTA{$SequenceName};
      chomp $Sequence;
      @Sequence = split("", $Sequence);

      my $LB = $Sequence[$LBP-1];
      my $RB = $Sequence[$RBP-1];

      $LB = $LB . $RB;
      $LB = uc $LB;

      if ($LB =~ /AA/g)  { $AA++; }
      if ($LB =~ /AG/g)  { $AG++; }
      if ($LB =~ /AC/g)  { $AC++; }
      if ($LB =~ /AU/g)  { $AU++; }
      if ($LB =~ /GA/g)  { $GA++; }
      if ($LB =~ /GG/g)  { $GG++; }
      if ($LB =~ /GC/g)  { $GC++; }
      if ($LB =~ /GU/g)  { $GU++; }
      if ($LB =~ /CA/g)  { $CA++; }
      if ($LB =~ /CG/g)  { $CG++; }
      if ($LB =~ /CC/g)  { $CC++; }
      if ($LB =~ /CU/g)  { $CU++; }
```

```
        if ($LB =~ /UA/g)  { $UA++; }
        if ($LB =~ /UG/g)  { $UG++; }
        if ($LB =~ /UC/g)  { $UC++; }
        if ($LB =~ /UU/g)  { $UU++; }

    }

    print
"$LBP\t$RBP\t$GC\t$CG\t$AU\t$UA\t$GU\t$UG\t$GA\t$AG\t$AA\t$GG\t$UU\t$CC\t$AC\t$UC\t$CA\t$CU
\n";

}

## BasePair is a subroutine that returns the basepairing pattern of an
## RNA inputted in bracket notation.

sub BasePair {

my @Struc = @_;
my $len = @Struc;

#Initiate two arrays to hold the basepair table, the two are synchronized
my @RtBracket = ();
my @LtBracket = ();

#This array keeps track of how the bps are related via bond-order
my @BondOrder = ();

#initiate a counter for the bonding scheme.
my $BoCount = 0;

#loop through the structure and match the left and right brackets increment the bond order
#for left brackets and decrement for right brackets. this keeps track of the position the first
#L bracket matches the last R bracket and so on.matching Bo #s gives bping pattern.
#single stranded bases are indicated with 0.

for (my $i=0; $i<$len; $i++) {
    if ($Struc[$i] eq "(") {
        #$LtBracket[$BoCount] = $i;
        $BoCount++;
        push(@BondOrder, $BoCount);
    }
    elsif ($Struc[$i] eq ")") {
        #$RtBracket[$BoCount-1] = $i;
         push(@BondOrder, $BoCount);
         $BoCount--;

    }
    else {
        push(@BondOrder, 0 );
    }
}
#Scan through the bonding scheme and generate a bp table
my @BasePairs = ();
my $length = @BondOrder;
my $Test = "";

#print @BondOrder;

#Loop through bo array and pick matching elements to add positions to bp array.
```

```
#Set elements to 0 after scan to avoid double counting!
for (my $j=0; $j<$length; $j++) {
    if ($BondOrder[$j] != 0) {
    my $Test = $BondOrder[$j];
    push(@BasePairs, $j + 1);
    $BondOrder[$j] = 0;
    for (my $k=0; $k<$length; $k++) {
        if ($BondOrder[$k] == $Test) {
            push(@BasePairs, $k + 1);
            $BondOrder[$k] = 0;
            }
        }
    }
}

return (@BasePairs);

}
```
